# Facile preparation of aqueous-soluble fluorescent polyethylene glycol functionalized carbon dots from palm waste by one-pot hydrothermal carbonization for colon cancer nanotheranostics

**DOI:** 10.1038/s41598-022-14704-x

**Published:** 2022-06-22

**Authors:** Amornrat Sangjan, Suthida Boonsith, Kanokwan Sansanaphongpricha, Tapanee Thinbanmai, Sakhon Ratchahat, Navadol Laosiripojana, Kevin C.-W. Wu, Hyeon Suk Shin, Chularat Sakdaronnarong

**Affiliations:** 1 Department of Chemical Engineering, Faculty of Engineering, Mahidol University, 999 Putthamonthon 4 Road, Salaya, Putthamonthon, 73170 Nakorn Pathom Thailand; 2 National Nanotechnology Center (NANOTEC), National Science and Technology Development Agency (NSTDA), 111 Thailand Science Park, Phahonyothin Road, Khlong Nueng, Khlong Luang, 12120 Pathum Thani Thailand; 3 The Joint Graduate School of Energy and Environment (JGSEE), King Mongkut’s University of Technology Thonburi, 126 Pracha Uthit Road, Bang Mot, Tungkru, 10140 Bangkok Thailand; 4 Department of Chemical Engineering, National Taiwan University, No.1, Sec.4 Roosevelt Road, Taipei, 10617 Taiwan; 5 Center of Atomic Initiative for New Materials (AI-MAT), National Taiwan University, Taipei, 10617 Taiwan; 6 International Graduate Program of Molecular Science and Technology, National Taiwan University (NTU-MST), Taipei, 10617 Taiwan; 7 Department of Energy Engineering, Ulsan National Institute of Science and Technology (UNIST), Ulsan, 44919 Republic of Korea; 8 Department of Chemistry, UNIST, Ulsan, 44919 Republic of Korea; 9 Center for Multidimensional Carbon Materials, Institute of Basic Science (IBS), Ulsan, 44919 Republic of Korea; 10 Low Dimensional Carbon Material Center, UNIST, Ulsan, 44919 Republic of Korea

**Keywords:** Nanoparticles, Synthesis and processing, Biomedical engineering, Chemical engineering

## Abstract

Carbon dots (CDs) are categorized as an emerging class of zero-dimension nanomaterials having high biocompatibility, photoluminescence, tunable surface, and hydrophilic property. CDs, therefore, are currently of interest for bio-imaging and nano-medicine applications. In this work, polyethylene glycol functionalized CDs (CD-PEG) were prepared from oil palm empty fruit bunch by a one-pot hydrothermal technique. PEG was chosen as a passivating agent for the enhancement of functionality and photoluminescence properties of CDs. To prepare the CDs-PEG, the effects of temperature, time, and concentration of PEG were investigated on the properties of CDs. The as-prepared CDs-PEG were characterized by several techniques including dynamic light scattering, high-resolution transmission electron microscopy, X-ray photoelectron spectroscopy, fluorescence spectroscopy, Raman spectroscopy, Fourier-transform infrared spectroscopy and Thermogravimetric analysis. The as-prepared CDs under hydrothermal condition at 220 °C for 6 h had spherical morphology with an average diameter of 4.47 nm. Upon modification, CDs-PEG were photo-responsive with excellent photoluminescence property. The CDs-PEG was subsequently used as a drug carrier for doxorubicin [DOX] delivery to CaCo-2, colon cancer cells in vitro. DOX was successfully loaded onto CDs-PEG surface confirmed by FT-IR and Matrix-Assisted Laser Desorption/Ionization Time-of-Flight Mass Spectrometer (MALDI-TOF/MS) patterns. The selective treatment of CDs-PEG-DOX against the colorectal cancer cells, , relative to normal human fibroblast cells was succesfully demonstrated.

## Introduction

Carbon dots (CDs) are a new class of small fluorescent nanomaterials with an average size below 10 nm^1^. The composition of core structure is mainly composed of carbon surrounded or embedded by heteroatoms and functional groups depending on synthesis techniques^2^. These carbon nanomaterials have been explored for their good biocompatibility, low toxicity, stable photoluminescence, water-solubility, multi-color fluorescence, high surface area, and excellent optical properties. CDs have found in broad applications, e.g. bioimaging, photocatalysis, drug delivery, chemo-sensing, bio-sensing, and solar cells. Several synthesis methods of CDs have been developed with various strategies such as electrochemical oxidation, chemical oxidation, laser ablation, pyrolysis, hydrothermal carbonization, and microwave irradiation^3^. After carefully evaluating the existing literatures, it has been concluded that several techniques and types of materials influence the yield and properties of synthesized CDs. Among them, hydrothermal synthesis is a potential means of CDs synthesis that can be scaled up to industrial-scale production. Various precursors such as orange peel, mango peel, wheat straw, milk, algae, citric acid, folic acid, urea, glycerol and agricultural wastes were reported as CDs precursors. However, CDs produced from a traditional hydrothermal carbonization method have a low quantum yield. To solve this problem, the developed one-pot synthesis of surface passivated CDs was introduced to enhance their fluorescence properties and functional groups for utilization as nano-carrier of targeted molecules^4^.

Several passivating agents were applied for the modification of CDs for enhanced their photoluminescent property, such as polyethylene glycol [PEG] and polyethyleneimine [PEI]. The attachment of heteroatom moieties onto the surface of CDs has been found to increase the fluorescent emission of CDs. Treatment with PEG, so called PEGylation, is the one of attractive approaches for preparation of passivated CDs. This polymer is non-toxic, non-immunogenic, non-antigenic, water soluble and easily conjugated to other biomolecules^5^. For instance, PEG-2000 was used as a surface modifier to synthesize PEG passivated CDs with the enhanced quantum yield, while -OH groups were substantially present on the CDs surface^6^. Shen et al*.* reported a successful one-pot hydrothermal synthesis of CDs from graphene and PEG-10000 for photoelectrode application. The CDs-PEG photoelectrode showed the small photocurrent under near infrared (NIR) laser and higher fluorescence properties compared to unmodified CDs^7^. Campos et al*.* demonstrated the hydrothermal synthesis of a fluorescent carbon dot using D-lactose as a carbon source and PEG-3350 as a coating material for producing a thermo-response microgel. CDs-PEG were encapsulated within microgels. The synthesized-CDs had a spherical shape with an average size of ~ 4 nm. The hydroxyl groups of PEG were successfully grafted on the CDs with PEGylation process^8^. Ruan et al*.* studied the effect of different modifiers for CDs synthesis and found that the quantum yield of CDs-PEG were effectively enhanced^9^.

Recently, CDs have been successfully employed as a nanocarrier and bioimaging agent to deliver drugs or a specific molecule to cancer cells. It has been reported that chemo-drug epirubicin^10^ entrapped in anionic CDs and cationic dendrimers synthesized from acetylated G5 poly(amido amine) (G5-Ac85), named as CDs@EPI⊂G5-Ac85 hybrids, induced MCF-7 breast cancer cell apoptosis^11^. Another chemotherapeutic drug that has been widely used for cancer treatment is doxorubicin^12^. Nevertheless, it has many disadvantages, including low cell internalization, low permeation and retention^13^, and cytotoxicity to normal cells^14^. To overcome these limitations, multifunctional nanocarriers for tumor-targeted drug deliveries have been developed to promote tumor accumulation of the drugs by using the enhanced permeability and retention effect^13^. Yang et al*.* constructed a nucleus-targeted drug delivery system based on the covalent conjugation of DOX and CDs functionalized with nuclear localization signal peptide (NLS-CDs) to improve its antitumor activity^15^. In another study, some researchers fabricated a nanocarrier for DOX-conjugated with βcyclodextrins (β-CD/CDs), which have ability to target folate receptor-positive cells. Their results proved the significantly increased the intracellular uptake and prolonged the release of DOX^16^.

From the literature review, therefore, in our present study, CDs-PEG was synthesized using one-pot hydrothermal carbonization, and oil palm empty fruit bunch (EFB) was chosen as carbon source due to its low cost. To develop a facile and cost-effective method, the simultaneous carbon dot formation and passivation was proposed to perform in one step. The effect of temperature and time for CDs and CDs-PEG synthesis was investigated. An optimal DOX loading on CDs-PEG and the controlled release were studied. The modified CDs-PEG were evaluated for their cytotoxicity and used as DOX nanocarrier toward CaCo-2 colon cancer cell delivery.

## Results and discussion

### Effect of temperature and time of hydrothermal carbonization for CDs synthesis

**Figure 1:**
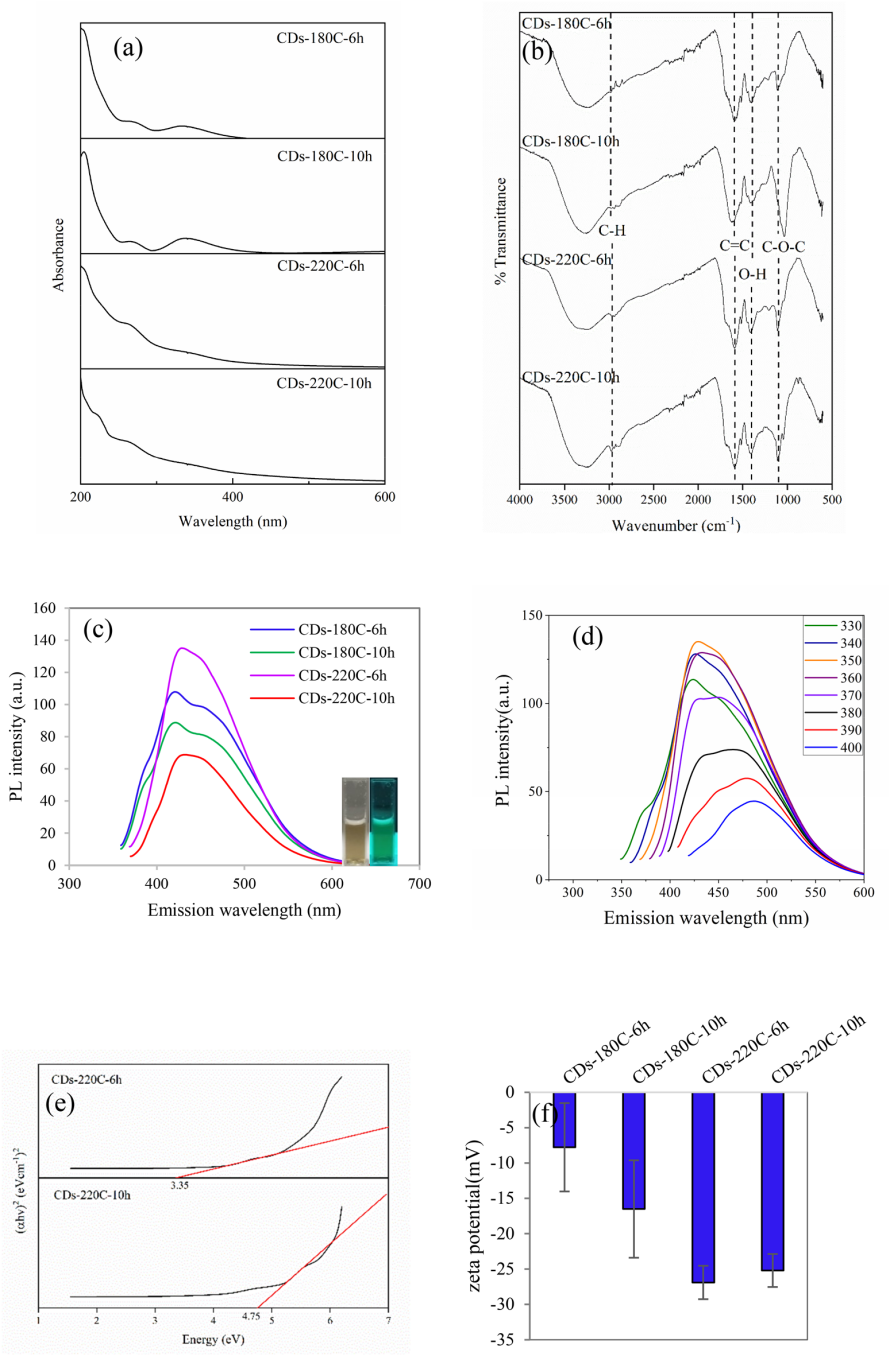
(**a**) UV-Vis spectra of CDs samples, (**b**) The FT-IR spectra of CDs samples, (**c**) Fluorescence emission spectra when the excitation wavelength was 340 nm for CDs-180C and 350 nm for CDs-220C. The upper-left corner inset shows image of CDs solution under daylights (left) and excited via UV light lamp (right), (**d**) PL spectra of CDs-220C-6h, (**e**) Energy band gap of CDs-220C, and (**f**) zeta potential of CDs synthesized from hydrothermal method at different temperatures and times.

In the present work, CDs were synthesized from EFB. After the hydrothermal reaction and purification, the brownish solution was obtained, in which the CDs were dispersed in the aqueous solution. In the first part, the effect of temperature (180 °C and 220 °C), and time (6 h and 10 h) of hydrothermal carbonization for CDs synthesis was studied. The synthesized products have been named as CDs-180C-6h, CDs-180C-10h, CDs-220C-6h, and CDs-220C-10h, respectively. UV-Vis spectroscopic analysis of as-prepared CDs was performed to characterize chromophore and nanodots characteristic. It was revealed that the optical absorption of nanomaterials of light sources, particularly nanodots or quantum dots, is significantly influenced by the monodispersity of the nanomaterials^17^. As shown in Fig. 1a, at low synthesis temperature (180 °C), high and obvious peaks of aromatic lignin chromophore at ~ 280 and ~ 315 nm were observed due to incomplete carbon dots formation. This was possibly attributed to complex electron transition on the surface, the π conjugated aromatic system, and the n–π* transition of the carbonyl and other oxygen-containing groups of lignin. It was additionally reported that absorption at 280 nm was due to non-conjugated phenolic lignin while that at 315 nm represented conjugated phenolic lignin units^18,19^. From Fig. 1a, the characteristic peak became broader and weaker when the reaction temperature was increased to 220 °C, as the total energy is reduced from the complete conjugated system and thus the stability of CDs was enhanced^20^.

The functional groups of all synthesized CDs samples at different temperatures and times were shown in Fig. 1b. All CDs sample showed uniform major FT-IR peaks at 3331 cm^−1^, 2975 cm^−1^, 1594 cm^−1^, 1404 cm^−1^, and 1109 cm^−1^. The peak around 3300 cm^−1^ (in the range of 3500–3000 cm^−1^) indicated the OH or NH stretching. Peaks appearing at 2900 cm^−1^ were assigned to CH stretching. The typical absorption band of C=C vibration could be observed around 1600 cm^−1^ while the peak at 1400 cm^−1^ revealed the OH group, and the peak at 1100 cm^−1^ was corresponded to C–O–C group^21^. For the photoluminescent property of CDs, Fig. 1c demonstrated the maximum PL intensity at the most suitable excitation wavelength of as-prepared CDs. The results showed that the emission wavelengths of all as-synthesized CDs from the hydrothermal synthesized CDs-180C-6h, CDs-180C-10h, CDs-220C-6h, and CDs-220C-10h were in the same range between 421 and 432 nm representing blue emitting CDs under 340–350 nm excitation wavelength. An increase of synthesis temperature significantly enhanced their maximum PL intensity since the hydrothermal process under lower temperature mainly involved dehydration, polymerization, and aromatization, while carbonization process occurs at the higher temperature^22^. Therefore, at increasing temperatures, a complete carbonization reaction to obtain CDs is achieved with a more graphitic carbon over amorphous structure, leading to the enhanced fluorescence intensity^20^. Raman spectroscopy for the CDs samples from different temperatures 180 °C and 220 °C for 6 h was shown in Fig. S1, exhibiting the intense peaks at 1379 cm^−1^ and 1567 cm^−1^ for D and G bands, respectively. The I_D_/I_G_ ratio of CDs-180C-6h was 0.79 and the I_D_/I_G_ ratio of CDs-220C-6h was 0.65. The results demonstrated that more graphitic carbon to amorphous carbon ratio (lower I_D_/I_G_ ratio) was observed from higher synthesis temperature. An enhanced fluorescence intensity when increasing synthesis temperature was due to smaller particle size of CDs indicated by hydrodynamic diameter shown in Table S1. Additionally, lower amount of chromophore structure from amorphous moieties which caused high UV-Vis absorption around 350 nm was found when increasing temperature (Fig. 1a), resulting in  greater fluorescence emission intensity when excitation wavelength was near aforementioned absorption. Moreover, zeta potential (Table S1) demonstrated more negatively charged surface functionalization of CDs synthesized at higher temperature (220 °C) for both 6 h and 10 h. It is widely known that the PL properties of a material can be attributed to energy-level transition, the radiative recombination of electrons and holes, the surface energy traps, the interactions between electrons and holes, and their surrounding environment^23^.

In case of CDs-220C-6h, varying excitation wavelengths of CDs from 330 to 400 nm exhibited a small change of emission wavelength toward red-shift emission from 450 to 500 nm (Fig. 1d). It was reported that the maximum emission peak near 450 nm from 350 nm excitation wavelength is due to the intrinsic emission of sp^2^ carbon hexagons. In contrast, the emission peak near 500 nm from 350 nm excitation wavelength and beyond is attributed to the extrinsic emission owing to either sp^3^ carbon or the defects of CDs containing oxygen-containing functional groups^24^. This phenomenon is supported by C1s and O1s XPS peak analysis (Table S2) yielding the ratio of sp^2^/sp^3^ of 1.03. Similar work reported that oxygen-containing functional groups may create new energy states (extrinsic state) inside the band gap of CDs resulting in the red shift of PL emission^25^. The blue color emitted CDs were obtained from the synthesis condition at CDs-220C-6h corresponding to synthesized CDs from lignin with the maximum emission at ~ 475 nm when excitation wavelength was 440 nm^26^. The fluorescence property of CDs synthesized at high temperature and shorter time showed the high fluorescence intensity. From Fig. 1c, the similar result was described by Dong et al.^27^ when the heating temperature was increased to 230 °C, the rate of cleavage and oxidation of carbon substrate was significantly increased, and thus CDs with more enhanced fluorescence intensity were formed in a short time (1 h).

Additionally, the variation in the energy band gap of the nanostructure can be determined from the UV-Visible absorption analysis by employing Tauc Plot^28^. From Fig. 1e, the energy band gap of CDs-220C-6h and CDs-220C-10h were found in the range 3.40 eV to 2.50 eV, similar to organic semiconducting dots^29^. In the present study, the smaller energy gap of 3.35 eV was obtained from CDs-220C-6h while CDs-220C-10h had the greater energy band gap of 4.75 eV. The narrower energy band gap of CDs-220C-6h was due to the lower ratio of the sp^2^ and sp^3^ fractions relative to sp^2^/sp^3^ ratio of CDs-220C-10h (Table S2). This could be engineered by elaborate syntheses techniques. Energy band gap of CDs was reported to decrease owing to oxygen, nitrogen and sulfur dopants in a respective degree rendering red-shift emission of CDs^30^. In the present study, surface passivation by oxygen atom indicating defects on CDs-220C-6h surface that increase sp^3^ illustrated by XPS peaks as demonstrated in Table S3. This caused the reduction of the energy band gap additionally identified by FT-IR spectroscopy with oxygen containing functional groups such as hydroxyl, carbonyl and carboxyl moieties connected to the CDs. The physical surface state of CDs was explained using the zeta potential that showed the negative values for all as-prepared CDs indicating negatively surface charged due to the existence of hydroxyl and carboxyl functional groups on the CDs surface^31^ as well as the stability of CDs in an aqueous phase as illustrated in Fig. 1f. High stability of CDs-220C-6h and CDs-220C-10h was achieved indicated by the zeta potential greater than − 30 mV.

**Figure 2:**
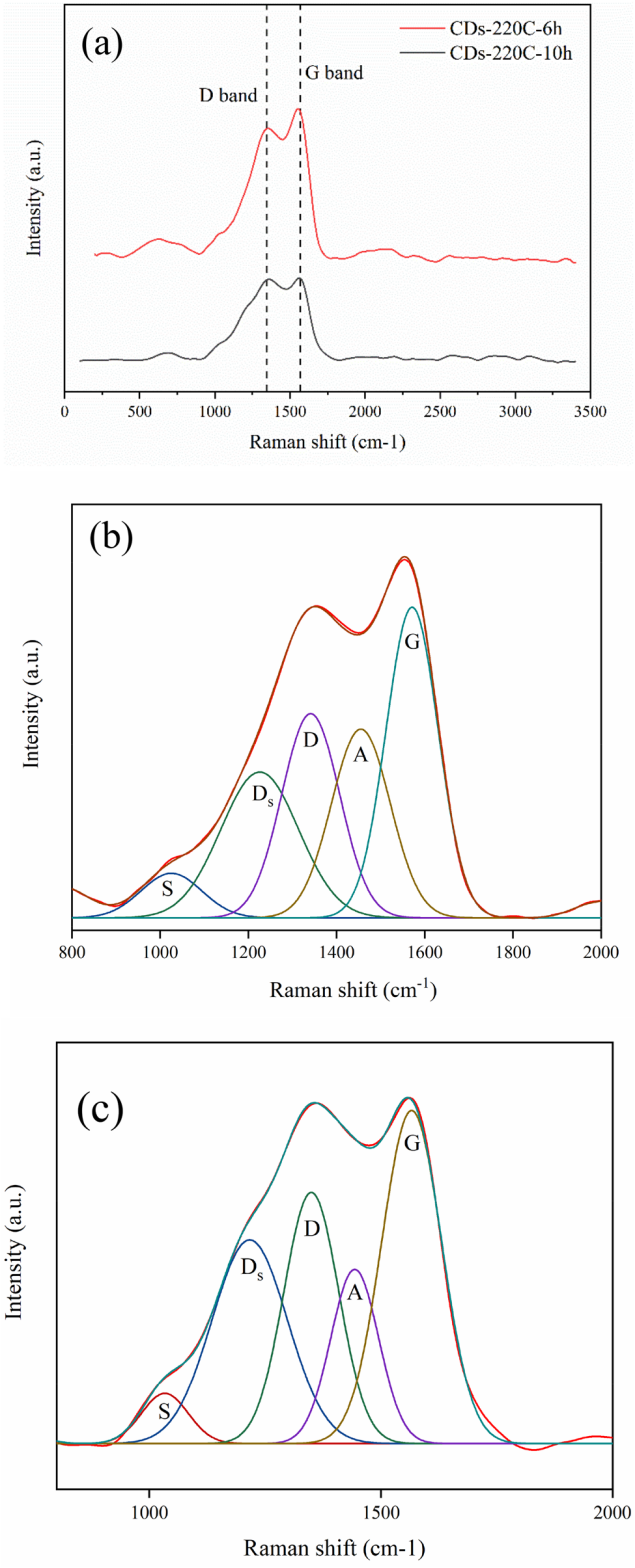
Raman spectra of (**a**) CDs-220C-6h and CDs-220C-10h, Deconvolution of Raman spectra of (**b**) CDs-220C-6h, and (**c**) CDs-220C-10h.

As demonstrated in Fig. 2, Raman spectroscopy of CDs-220C-6h showed two prominent peaks attributed to D band and G band. The CDs-220C-6h peak at 1346 cm^−1^ was assigned to disorder (D) band which is appeared due to the sp^3^ defects while the second peak at 1567 cm^-1^ was attributed to a crystalline (G) band which is related to the in-plane vibration of sp^2^ carbons^32^. The G band in Raman spectrum corresponds to the sp^2^ hybridization from graphitization associated in the CDs, and the D band illustrates the sp^3^ hybridization of carbon due to the contribution from the amount of edges and defects, and functionalization^33^. In case of CDs-220C-6h, Raman spectrum exhibited the intense peaks at 1346 cm^−1^ and 1573 cm^−1^ for D- and G-bands, respectively^34^. However, the Raman spectrum was not obvious in case of CDs-220C-10h. It was reported that the Raman characterization might be disturbed by the strong fluorescence of CDs^35^. In addition, the absence of the two peaks further proves that the CDs are composed of nanocrystalline graphite-like core and disordered sp^3^-carbon^36^. An increase of synthesis duration from 6 to 10 h gave a transformation of carbon structure between D and G bands indicating by a change of I_D_/I_G_ ratio. From Fig. 2, the I_D_/I_G_ ratio of CDs-220C-6h was 0.82 and the I_D_/I_G_ ratio of CDs-220C-10h was 0.92 exhibiting a decrease of crystalline G band of CDs-220C-10h and an increase of defect density or amorphous carbon as compared with sp^2^ hybridization in as-prepared CDs-220C-6h^32^. This corresponded to lower energy band gap of CDs-220C-6h. It has been reported that CDs synthesized from top-down method e.g. graphene gave higher Raman intensity and lower I_D_/I_G_ ratio compared with CDs synthesized from bottom-up method from natural carbon sources e.g. chitosan, collagen, humic substances, and plant seeds^32,33,37,38^ which mostly provide amorphous carbon nanostructures. Additionally, Fig. 2 b,c revealed the deconvolution of Raman spectra for A band between 1400 and 1460 cm^−1^ which represents breathing mode for 5-membered ring with Kekulé vibrations in adjacent 6-membered rings^39^, and heteroatom defects which tend to cause greater red shift. Secondary breathing modes for 7 + membered ring at 975–1075 cm^−1^ assigned to the symmetric breathing mode of various small polyaromatic hydrocarbons (PAH) as well as rings containing seven or more carbons represented as S band^40^ were detected for both CDs-220C-6h and CDs-220C-10h samples. From Fig. 2 b,c, an increase of synthesis time tends to alter 5-membered and 7-membered rings of both A and S bands to more stable 6-membered rings indicated by D and G bands.

**Figure 3:**
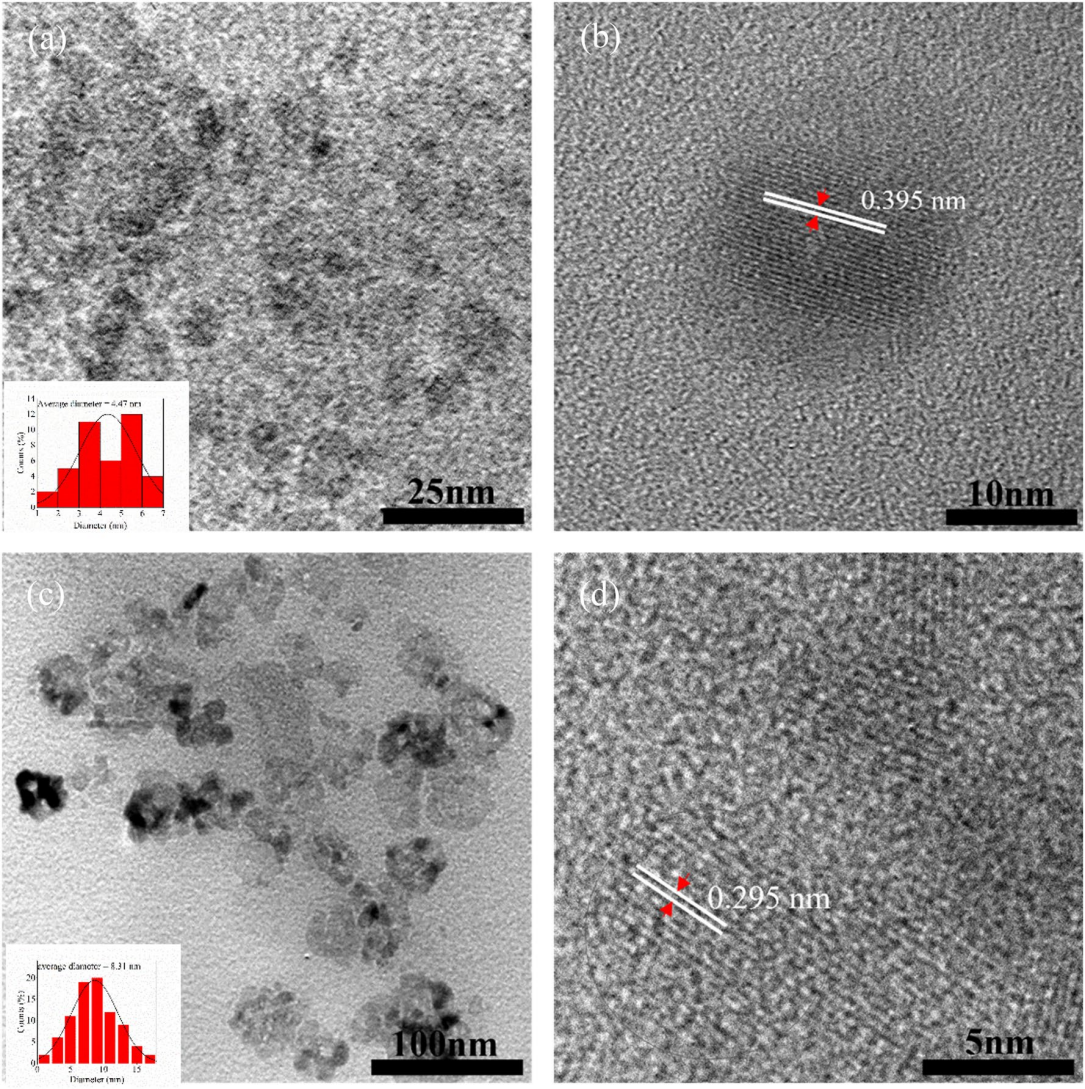
(**a**) HR-TEM images with size distribution histogram inset, (**b**) HR-TEM image of a typical CDs, showing the solidified graphite liner core of CDs at 220 °C for 6 h (CDs-220C-6h), (**c**) HR-TEM images with size distribution histogram inset, (**d**) HR-TEM image of a typical CDs, showing the solidified graphite liner core of CDs at 220 °C for 10 h (CDs-220C-10h).

HR-TEM was performed to observe the morphology of CDs-220C-6h and CDs-220C-10h. The results showed that CDs-220C-6h had spherical shape with an average diameter of 4.47 nm with D-spacing of 0.395 nm (Fig. 3a,b) while CDs-220C-10h showed the similar spherical morphology with an average size of 8.31 nm with D-spacing of 0.295 nm (Fig. 3c,d) which corresponded to (002) plane of graphitic carbon^35,41^. The D-spacing of this paper was calculated by using Fast Fourier Transform (FFT) image using Gatan Digital Micrograph Software, and the calculated average value of D-spacing with statistical analysis of image processing was shown in Fig. S2. The broader D-spacing of CDs-220C-6h than that of graphitic carbon was mainly because CDs-220C-6h consist of sp^2^ and sp^3^ hybridization based on Raman and XPS analysis, and thus the oxygen groups on the CDs-220C-6h surface might have enhanced the interlayer distance. The result was in agreement with a previous research^42^.

**Figure 4:**
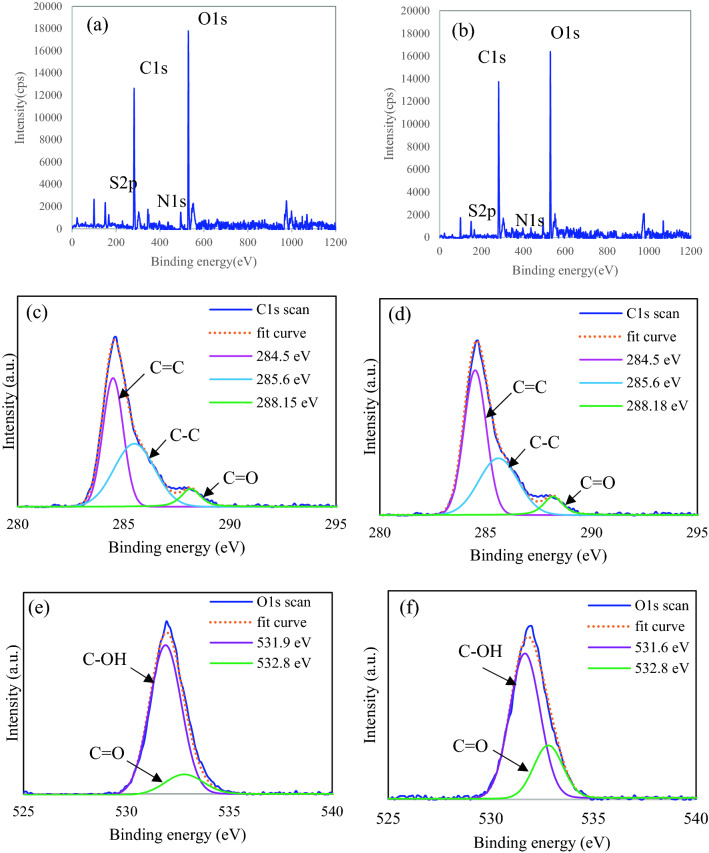
XPS spectroscopic survey of (**a**) CDs-220C-6h, and (**b**) CDs-220C-10h; C1s scan of (**c**) CDs-220C-6h, and (**d**) CDs-220C-10h; O1s scan of (**e**) CDs-220C-6h, and (**f**) CDs-220C-10h.

XPS analysis demonstrated elemental composition on the surface of CDs-220C-6h and CDs-220C-10h as shown in Fig. 4. The XPS survey spectrum (Fig. 4a,b) displayed two prominent peaks of oxygen (O1s at 531.4 eV) and carbon (C1s at 284.6 eV). These results suggested that the main elemental composition of CDs-220C-6h and CDs-220C-10h were carbon and oxygen. As shown in Fig. 4b, XPS spectrum corresponding to C1s of CDs-220C-6h showed the peaks at 284.5, 285.6, and 288.1 eV binding energy. The peak at 284.5 eV represents the hybridized carbon sp^2^ (C=C) and the hybridized carbon sp^3^-hybridized (C–C) within CDs carbon cores. The other bands of hydroxyl bound to C–O was found at 285.6 eV, and the binding energy at 288.1 eV attributed to carbonyl carbon C=O^21^. For the XPS spectrum corresponding to O1s of CDs-220C-6h, Fig. 4c showed XPS peaks at the binding energy of 531.6–531.9 and 532.8 eV, representing the structural oxygen of C–OH and C=O, respectively^43^. Similar XPS pattern was obtained from CDs-220C-10h.

In comparison with C1s and O1s contents of CDs-220C-6h and CDs-220C-10h, the peak area of XPS deconvolution was taken into consideration as demonstrated in Table S2 and Table S3. The results showed that the considerably higher O1s/C1s ratio represented as O/C atomic ratio was obtained from CDs-220C-6h relative to that from CDs-220C-10h. However, a substantial reduction of O/C of EFB (Table S4) compared with O/C ratio of all CDs samples (Table S3) indicates that carbonization occurred during hydrothermal synthesis of CDs. The data from all characterization techniques confirmed the sp^2^/sp^3^ hybridized carbon core structure of the synthesized CDs-220C-6h with intrinsically negatively charged functional groups attached to the surface of CDs-220C-6h that enabled them high colloidal stability in aqueous solution for further bio-applications.

### Characterization of PEG passivated on CDs (CDs-PEG)

**Figure 5:**
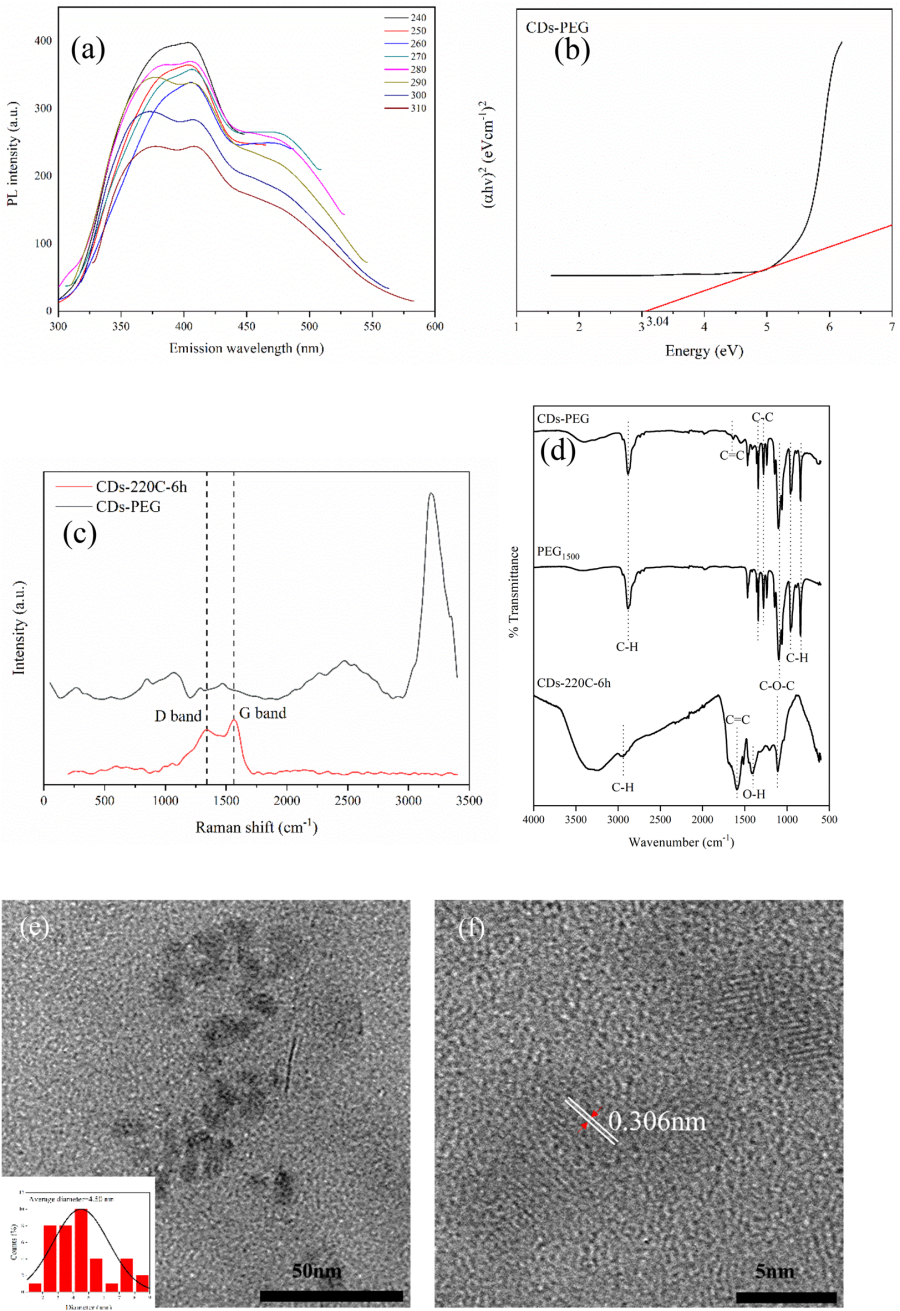
CDs-PEG characterization (**a**) PL spectra of CDs-PEG synthesized by hydrothermal carbonization at 220 °C for 6 h, (**b**) Energy band gap of CDs-PEG, (**c**) Raman spectroscopy of CDs-PEG and CDs-220C-6h, (**d**) FT-IR spectra of CDs-220C-6h, neat PEG1500 and CDs-PEG, (**e**) HR-TEM images with size distribution histogram inset, (**f**) HR-TEM image of a typical CDs, showing the solidified graphite liner core of CDs-PEG.

In this part, one-pot CDs with PEG passivation was synthesized at 220 °C for 6 h under hydrothermal condition for further application as drug nanocarrier toword cancer cells. As demonstrated in Fig. 5a, CDs-PEG showed the significant increase of PL intensity for approximately three times compared with CDs (Fig. 1c). The alteration of excitation wavelength from 350 to 240 nm that gave the maximum emission near 400 nm was due to an alteration of band gap energy of CDs-PEG which could be approximated by Tauc Plot. As demonstrated in Fig. 5b, the energy band gap of CDs-PEG was 3.04 eV which was smaller than that of CDs-220C-6h (3.37 eV). The narrower band gap was possibly caused by the coverage of OH and COOH and different dopant molecules. Previous reports revealed that LUMO − HOMO band gap of CDs gradually decreases as the coverage of OH and COOH on the surface increases^44^, and as a result of O, N, and S dopants in a respective degree^30^. Previous reports showed that PL intensity of CDs is mainly related to the trapping of excited-state energy of the surface-passivated CDs^5,7^. As a result, PEG passivation on CDs surface could stabilize the surface energy trap of CDs and enable them PL emissive with intensity three-time stronger than CDs without PEG passivation. The Raman peaks of CDs-PEG (Fig. 5c) at 1346 cm^−1^ and 1573 cm^−1^ for D- and G-bands, respectively were deminised in case of CDs-PEG compared with CDs-220C-6h. This was because the disturbance by the strong fluorescence of CDs-PEG^35^. Raman peak near 3200 cm^−1^ represented the complementary analysis of the Raman-active C–H stretching modes of PEG (3000–2800 cm^−1^) and the slight shift of Raman peak from 3000 to 3200 cm^−1^ was presumably due to a shorter chain of PEG1500 to PEG with monomer units between 1 and 9 units^45^.

To confirm the PEG surface passivation on CDs, FT-IR spectroscopic analysis was performed. As shown in Fig. 5d, the major peaks of PEG were shown in the CDs-PEG, therefore the result confirmed the successful surface passivation of PEG on CDs surface. CDs-220C-6h showed major FT-IR peaks at 3331 cm^−1^, 2975 cm^−1^, 1594 cm^−1^, 1404 cm^−1^, and 1109 cm^−1^. The peak around 3300 cm^−1^ (in the range of 3500–3000 cm^−1^) indicated the OH or NH stretching mode. Peaks appeared at 2900 cm^−1^were assigned to CH stretching vibration. The typical absorption band of C=C vibration could be observed around 1600 cm^−1^. The peaks at 1400 cm^−1^ revealed the OH group, and peaks at 1100 cm^−1^ corresponded to C–O–C group^21^. In case of CDs-PEG, FT-IR peaks at 2882 cm^−1^, 1340 cm^−1^, 1279 cm^−1^, 1092 cm^−1^, 959 cm^−1^, and 841 cm^−1^ were found. The aforementioned FT-IR peaks were appeared in pure PEG. The stretching vibration at 2880 cm^−1^ corresponded to C-H. The peaks at 1340 cm^−1^ and 1279 cm^−1^ were due to the C–C group^46^. The band at 1092 cm^−1^ was assigned to C–O–C^21^, and the peaks appeared at 800–900 cm^−1^ were attributed to aromatic C–H bond^5^. Moreover, plenty of hydrophilic groups for instance hydroxyl, carbonyl, and carboxyl groups found on the surface of CDs-PEG facilitated the particles with good solubility in the aqueous solution.

From the HR-TEM image analysis of CDs-PEG shown in Fig. 5e,f, the average core carbon size of CDs-PEG was 4.50 nm (lattice space = 0.306 nm) which was in the same range of CDs-220C-6h (4.47 nm). The core carbon diameter of CDs-PEG from HR-TEM was approximately 500 times smaller than hydrodynamic size diameter (2434.33 nm) from Table S1. The large hydrodynamic size of CDs-PEG ascribed was due to the existence of PEG and their interaction with water^47^. There are more possible explanations for the overestimated hydrodynamic size of nanoparticle in the solution from DLS technique^48^. Moreover, the PEG layer may form a corona structure in the aqueous solution, which contributes to the considerably enhanced dynamic light scattering of the nanoparticles, and moreover, the nanoparticles may entangle together due to strong hydrogen bonding and form reversible aggregation. The high mass yield was possibly due to one-pot synthesis of CDs-PEG technique in which PEG could become both carbon precursor and passivation agent for CDs formation during hydrothermal reaction at high temperature^49^.

**Figure 6:**
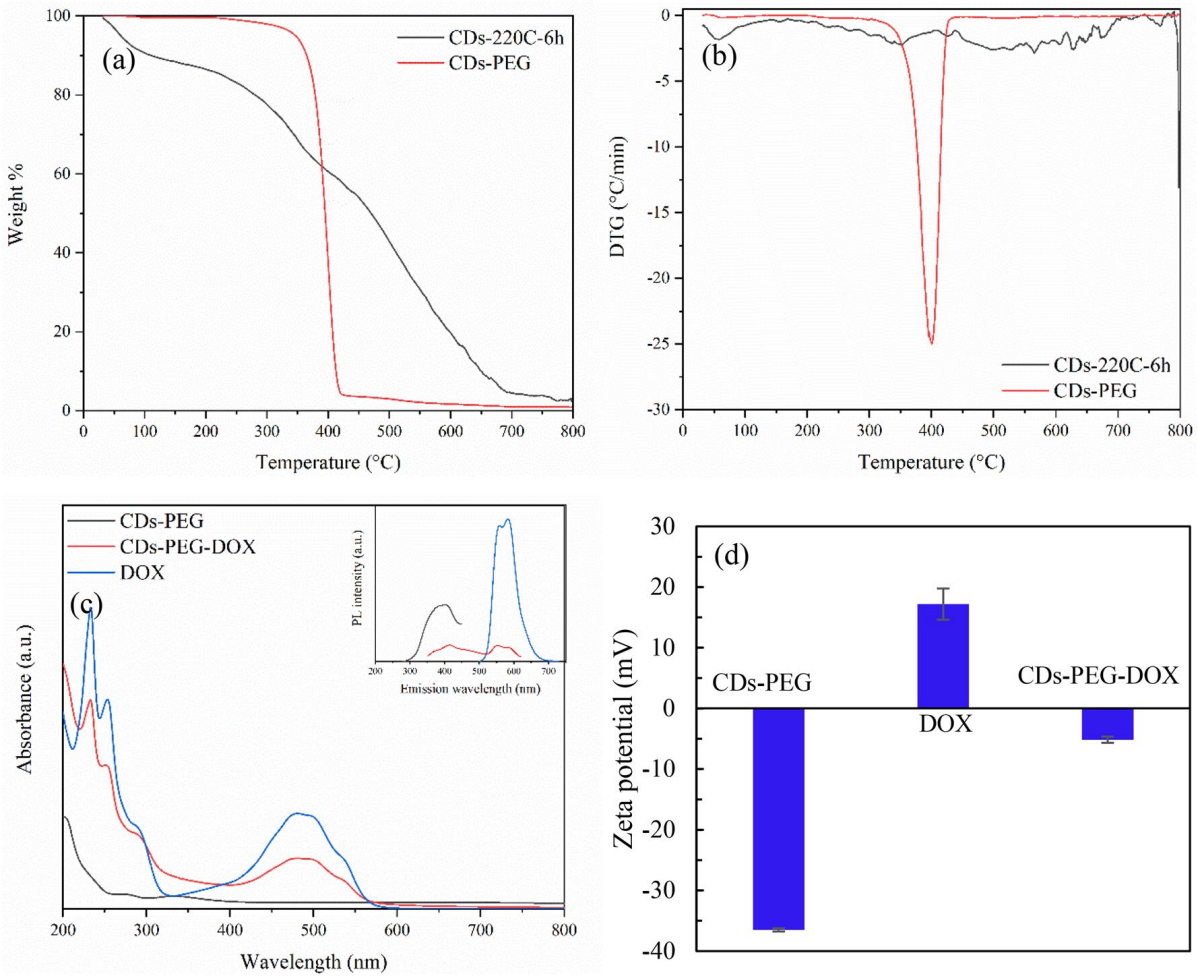
Thermal degradability of CDs-PEG and CDs-220C-6h; (**a**) TGA, (**b**) DTA curves under nitrogen atmosphere, (**c**) UV-Vis absorption spectra of CDs-PEG (220 °C, 6 h), DOX, and CDs-PEG-DOX. The inset shows fluorescence spectra of CDs-PEG (excitation at 240 nm), DOX (excitation at 480 nm), and CDs-PEG-DOX (excitation at 330 nm), and (**d**) the surface zeta potential of CDs-PEG, DOX, and CDs-PEG-DOX.

The XPS survey spectrum of CDs-PEG (Fig. S3(b)) displayed two prominent peaks of oxygen (O1s at 531.4 eV) and carbon (C1s at 284.6 eV). As shown in Fig. S3(d), carbon atom analysis of C1s showed the peaks at 283.1 and 285.6 eV binding energy attributed to π-C interaction and C–C vibration. Compared with CDs-220C-6h, the disappearance of C=O and C–C peaks at 288.18 eV and 285.6 eV was observed in case of CDs-PEG. This was possibly due to the hydrogen substitution from PEG molecules during hydrothermal carbonization. The peaks at 283.1 eV represents the π-bonded carbon atoms. The other bands of hydroxyl bound to C-O was found at 285.6 eV^21^. For the spectrum of the oxygen atom O1s, Fig. S3(f) showed the XPS peak at the binding energy of 530.5 and 531 eV, representing the structural oxygen in the structure of C=O, C–OH and C–O–C components^43^. Moreover, the O/C atomic ratios were 0.52 and 0.38 for the CDs and CDs-PEG, respectively indicating the incorporation of ethylene glycol moieties bound to the surface of the CDs nanoparticles^8^. These results suggested that the main elemental composition of CDs-PEG were carbon and oxygen and PEG was covalently bound to CDs. Figures 6a,b show the thermal degradation of CDs and CDs-PEG synthesized by hydrothermal carbonization at the same condition at 220 °C for 6 h. It was observed that CDs-220C-6h showed the slow decomposition according to the heating time, however CDs-PEG showed the rapid degradation at temperature 400 °C regarding the breakdown of PEG molecules passivated on CDs. From the previous report, PEG (Mw = 6000) was thermally decomposed starting from 250 to 400 °C for complete decomposition of PEG^50^. With narrow DTG peak from CDs-PEG illustrated in Fig. 6b, the result indicated lower molecular weight PEG and narrow polydispersity of synthesized CD-PEGs.

### Characterization of DOX functionalization on CDs-PEG (CDs-PEG-DOX)

The UV-Vis absorption and fluorescence spectra of CDs-PEG, CDs-PEG-DOX and DOX were shown in Fig. 6c. The UV-Vis absorption peak of CDs-PEG was not obvious, but could be detected as small shoulders at 272 nm and 334 nm which were mainly attributed to π-π* electron transition of C=C bonds in CDs conjugated structure^51^. The UV-Vis absorbance of DOX shows a prominent peak at 480 nm. After DOX was functionalized onto CDs-PEG, the UV-Vis absorption of CDs-PEG-DOX displayed both of CDs-PEG and DOX absorption peaks. The absorption peaks of CDs-PEG at 272 and 334 nm were shifted upward, and major peak of DOX in CDs-PEG-DOX at 480 nm was still intense. When photoluminescence property is considered, the CDs-PEG showed a strong PL emission intensity at 403 nm under the excitation at 240 nm (inset). The PL emission intensity of DOX was notable at the characteristic peaks of 554 nm and 583 nm under excitation at 480 nm. However, the fluorescence intensity of CDs-PEG-DOX decreased when compared with CDs-PEG. It has been revealed that the overlapping between CDs-PEG emission and DOX absorption at 240 nm conceivably generated fluorescence resonance energy transfer process and thus interfered PL emission intensity of CDs-PEG-DOX^52^. The zeta potential shown in Fig. 6d additionally proved the binding between CDs-PEG and DOX. From the results, the zeta potential of CDs-PEG and DOX were − 36.5 and + 17.2 mV, respectively. After functionalization, the zeta potential of CDs-PEG-DOX was − 5.16 mV which was ascribed to the occurrence of charges neutralization between CDs-PEG and DOX.

To confirm the binding of DOX onto the functional groups of CDs-PEG, the MALDI-TOF-MS spectra of CDs-PEG, DOX, and CDs-PEG-DOX with α-cyano-4-hydroxycinnamic acid (CHCA) matrix were analyzed. As shown in Fig. S4, CDs synthesized from 220 °C at 6 h and 10 h exhibited interference-free background in the mass range of m/z 25–850 indicating that CDs are very suitable for the analysis of small molecules with MALDI-TOF-MS^53^. In the present study, MALDI-TOF-MS analysis in negative mode demonstrated detection of small anion molecules at m/z 26, 50, 72, 93, 144, 189 which were corresponding to the signals of carbon cluster anions from C_1_^–^ to C_10_^–^ that can be detected in negative-ion mode at 12, 24, 36, 48, 60, 72, 84, 96, 108, and 120 from previous works^54^. These data confirmed the structure of CDs-220C-6h and CDs-220C-10h from XPS analysis and revealed that the as-prepared CDs were functionalized with –COOH and/or –OH groups, which made it possible for liberating and transferring proton to analyte in positive ion MALDI-TOF-MS^55^. The signals in positive-ion mode is unclear, however it can be possibly explained by the cause of ionization of adsorbed hydrocarbon contaminants or the fragmentation of carbon structure. These phenomena were also observed in porous silicon and graphene matrices^53,56^.

In summary, to detect PEG and DOX on CDs particles, MALDI-TOF-MS which is an efficient technique to detect small molecules was used. It has been reported that PEG with different molecular weights could effectively analyzed by MALDI-TOF-MS for positive mode due to protonation of PEG molecules during laser ionization^57^. In MALDI-TOF-MS analysis of PEG, CHCA was reported as the best matrix to improved homogeneity of sample surface in positive ion mode measurement^58^ while other solvents e.g. 9-aminoacridine (9-AA) are suitable matrices for negative mode^59^. However, signal to noise ratio (S/N ratio) of spectral data was found to be slightly increased when adding CHCA matrix. Even though, many reports showed evidences of noise suppression of using nanoparticles as a matrix to replace organic solvent. However, most carbon material matrices exhibit low solubility and dispersibility in solution, resulting in easy aggregation, low reproducibility, and heterogeneous crystallization with analytes^60^. Therefore, the aggregated CDs possibly contaminated the ion source. After CDs was functionalized with PEG, there were cation molecules found at m/z 1000–2000 indicating the functionalized PEG on CDs surface. Conjugation of DOX onto CDs-PEG particles was confirmed by MALDI-TOF-MS at m/z 396 and 399 in negative-ion mode as demostrated in Fig. S4 (K and L) which was in good accordance with mass spectroscopic analysis results^61,62^.

### DOX loading and release efficiency of CDs-PEG-DOX and in vitro cytotoxicity test

From the DOX loading study, the optimal CDs-PEG:DOX ratio of 10:1 (1 mg mL^−1^ CDs-PEG to 100 μg mL^−1^ DOX) was found to yield the maximum loading efficiency^63^ and maximum loading content (DLC) of DOX onto CDs-PEG was 94.6% and ~ 95 mg g^−1^, respectively (Table S5). The driving force of DOX loading is surface charge difference between negatively charged CDs-PEG (− 36.5 mV) and positively charged DOX (+ 17.2 mV) expressed by zeta potential demonstrated in Fig. 6d.

In certain cancers, particularly cancers of kidney, brain, lung, breast, and colon, there are evidence of folic acid (FA) accumulates due to over-expression of FA receptors on their surfaces^64^. Owing to solid tumor’s biochemical properties e.g. distorted extracellular matrix and fuzzy layer of tumors as well as acidified environment^65^, drug delivery to tumors becomes intimidating task. In case of CDs-PEG on DOX delivery, we found that an enhanced rate of DOX release was triggered by pH. In acidic environment (pH 5.0 at 37 °C), DOX release from CDs-PEG-DOX was rapidly at the beginning within 30 h, and reached the equilibrium state afterward at maximum DOX release of 23%. Under acidic condition, the hydrophobic bond of *π*–*π* interactions between CDs-PEG and DOX were opened, and DOX was released^66^. Then the electrostatic forces between PEG and DOX were disrupted due to the protonation of the primary amine functional group of DOX. DOX was subsequently released from the nano-carrier^67,68^. However, the release of DOX remained low after 48 h (Fig. S5) which was probably due to strong hydrophobic interaction between the CDs and DOX. Slower rate of DOX release was obtained at pH 7.4 and reached the maximum DOX release of 36% at 4 days. This dual sensing drug delivery system provides beneficiary effect on cancer chemotherapy since there are cells in solid tumors that maintain both acidic and basic pH depending on their spatial distribution from blood vessel^69^. Therefore, CDs-PEG conjugated with DOX can play a role in combating most of cell types of colon cancer cells^70^.

**Figure 7:**
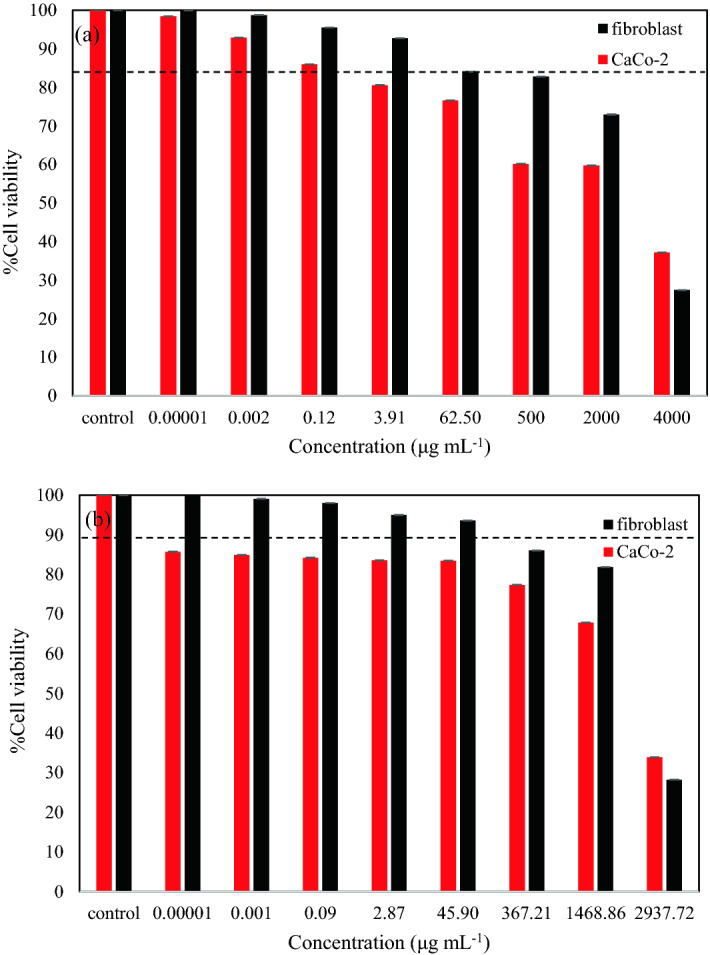
Cytotoxicity of fibroblast and CaCo-2 cells for 24 h incubation at different concentrations of (**a**) CDs-PEG, (**b**) CDs-PEG-DOX from 0.1–0.2 ng mL^−1^ to 2.9–4.0 mg mL^−1^.

**Figure 8:**
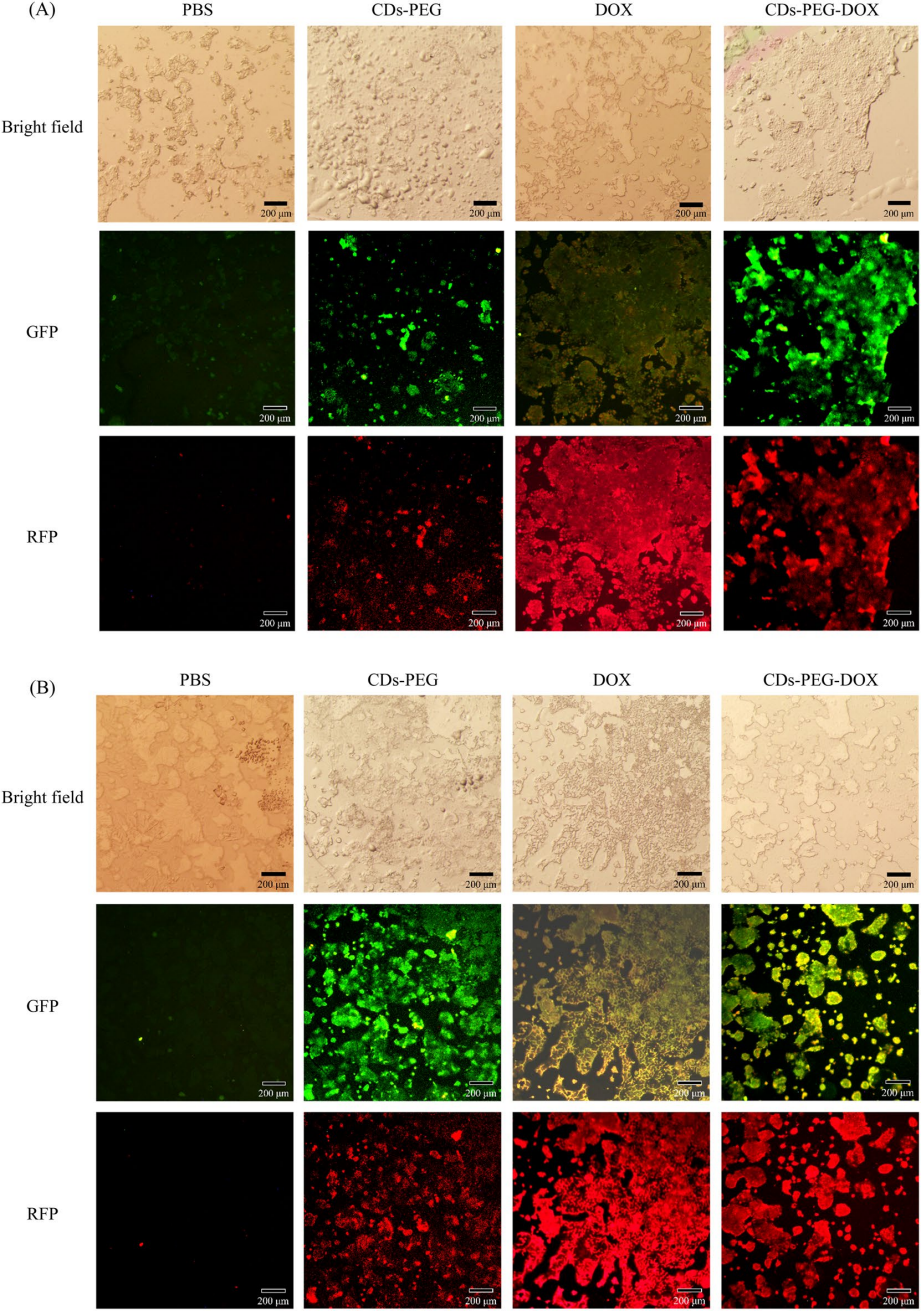
Fluorescence microscopic images of CaCo-2 cells after incubation in PBS, CDs-PEG, DOX, and CDs-PEG-DOX synthesized from 3 mg mL^−1^ of CDs-PEG containing 300 μg mL^−1^ DOX for (**A**) 1 h, and (**B**) 5 h. (GREEN—CDs-PEG, RED—Doxorubicin). Scale bar: 200 μm. Filters : (GREEN—CDs-PEG, RED—Doxorubicin) when Green Fluorescent CDs-PEG label was detected at λ_ex_ = 420–490 nm (GFP filter) and DOX label at λ_ex_ = 510–560 nm (RFP filter).

To determine the efficacy of CDs-PEG-DOX against the colon cancer cell, the cytotoxicity test CDs-PEG and CDs-PEG-DOX was evaluated using WST-1 assay. Colon cancer cells (CaCo-2) and Fibroblast were chosen as the cancer model and the normal cell control, respectively. From recent studies, there was clear evidence that cancer-associated fibroblasts (CAFs) play a crucial role in intestinal tumor^71^ as well as colon cancer cells progression^72^. Fibroblasts were found to surround tumor cells and consist of various heterogeneous subsets which can exert both tumor-promoting and -suppressing functions^73^, hence they were selected as normal cell control in the present study. As demonstrated in Fig. 7a, fibroblast cells maintained high cell viability over 85% until the concentration of in CDs-PEG at 3.9 μg mL^−1^, and the concentration higher than this level can cause toxicity to fibroblast cells. In contrast, CDs-PEG can selectively cause significant toxicity toward CaCo-2 cells at very low concentration of 0.12 μg mL^−1^. After being functionalized with DOX as shown in Fig. 7b, the CDs-PEG-DOX exhibited that CaCo-2 cells viability was significantly lower than that of CDs-PEG. The cell viability of CaCo-2 cells decreased to lower than 85% at low CDs-PEG-DOX concentration at 0.011 μg mL^−1^ (11 ng mL^−1^), while high viability of fibroblast over 85% was maintained at high concentration of CDs-PEG-DOX at 370 μg mL^−1^. It is probably because CaCo-2 cells have higher cellular uptake of the nanoparticles than normal fibroblast cells. Substantial reduction of cell viability lower than 50% was observed at 2,940 μg mL^−1^ CDs-PEG-DOX. The average data and S.D. were additionally reported in Table S6 and the results indicated excellent anticancer effect of CDs-PEG-DOX toward CaCo-2 cells. Results of microscopic evaluation of primary fibroblast cells and CaCo-2 cells after treated with CDs-PEG and CDs-PEG-DOX that confirmed the decrease of cell viability were illustrated in Figs. S6–S10. In addition, the results of CaCo-2 cell uptake of CDs-PEG and CDs-PEG-DOX showed good tumor cell uptake within 5 h as illustrated in Fig. 8. Different emission intensities at distinct excitation wavelength of CDs-PEG and DOX indicated their suitability for in vivo applications of anticancer drug delivery and monitoring.

Additionally, the in vitro cytotoxicity test of control sample of CDs was conducted and compared with CDs-PEG and CDs-PEG-DOX for mouse fibroblast cells (L929) and human colorectal adenocarcinoma cells (HT-29) as shown in Table S7–S8 and Figs. S11–S17. It was found that L929 was inhibited for IC_50_ at 35.81 μg mL^−1^ by CDs-PEG-DOX and no inhibition was observed for HT-29 at 500 μg mL^−1^. Substantially less inhibition was found for CDs for both L929 (IC_50_ = 637 μg mL^−1^) and HT-29 (IC_50_ = 1177.76 μg mL^−1^). Interestingly, when coating CDs with PEG, no inhibition of CDs-PEG was observed on both HT-29 and L929 cells even at highest concentration of CDs-PEG at 5000 μg mL^−1^.

The stability test of CDs, CDs-PEG and CDs-PEG-DOX confirmed the covalent bonding between CDs and PEG. The zeta potential (Fig. S18a) and UV-Vis absorbance of CDs and CDs-PEG (Fig. S19a and S19b) when stored in darkness at 4 °C for 1 and 2 weeks did not change substantially compared with as-prepared samples, however the UV-Vis absorbance of DOX at 480 nm disappeared after 1 week storage (Fig. S19(c)). The hydrodynamic diameters of all samples (CDs, CDs-PEG and CDs-PEG-DOX) slightly increased after 1 week and constant from 1-week to 2-week storage (Fig. S18b). Fluorescence intensity of CDs considerably decreased within 1 week and then constant while that of CDs-PEG gradually decreased from as-prepared to 2-week storage (Fig. S18c and S18d, respectively). In contrast, the fluorescence intensity of CDs-PEG-DOX was quenching for as-prepared sample from the adsorption of DOX molecules onto CDs-PEG through electrostatic force, while 1-week and 2-week storage caused detachment or degradation of DOX and thus fluorescence property of CDs-PEG markedly increased (Fig. S18e).

## Conclusions

In summary, CDs were successfully prepared under hydrothermal carbonization reaction using EFB as a precursor. Moreover, CDs were directly surface passivated by PEG by facile one-pot synthesis enabling it greater luminescent, biocompatible and highly soluible in aqueous solution. The obtained CDs had a spherical shape with diameter of 4.47 nm mainly containing hybridized sp^2^/sp^3^ carbon nanostructure with oxygen-containing functional groups. The surface passivated CDs-PEG demonstrated blue emission property with strong fluorescence intensity, and plenty of hydrophilic groups suitable for further functionalization with doxorubicin, a model anticancer drug. CDs-PEG exhibited excellent ability as Doxorubucin nanocarrier toward CaCo-2 colorectal cancer cells indicated by effective inhibition to the proliferation of CaCo-2 cells due to pH-responsive DOX release in the cells. Due to their high biocompatibility, stability in aqueous phase, and simple synthesis method, CDs-PEG potentially serves as a new type of nanomaterial with good fluorescence properties for bio-related applications.

## Material and methods

### Materials

EFB was collected from Chumporn Palm Oil Industry Company Limited, Chumporn Province, Thailand. Polyethylene glycol 1500 (PEG 1500) was purchased from Alfa Aesar, UK. Doxorubicin hydrochloride was purchased from Tokyo Chemical Industry, Japan. Deionized water (~ 18.2 MΩ) was obtained from a water purifying system (Thermo scientific D7411, USA). Dialysis membrane (MWCO = 1000 Da) was purchased from Cole-parmer, USA.

### One-pot carbon dots synthesis and surface passivation

EFB was first washed by tap water and dried in sunlight, followed by milling and screening to the size of + 50/− 200 mesh. For the screening of suitable condition for CDs synthesis, 1.5 g EFB and 30 mL of deionized water were mixed in a Teflon-lined autoclave reactor having a working volume of ~ 70 mL. The hydrothermal carbornization was performed by heated the reaction mixture to the desired temperature (180 °C and 220 °C) for a certain time (6 h and 10 h). The effect of PEG functionalization was studied with the ratio of EFB to PEG of 1 : 3.3 by weight. After the hydrothermal carbonization reaction, the autoclave reactor was cooled down to room temperature and the obtained brown solution was centrifuged to separate liquid and coarse particles. The aqueous solution was then filtered through 0.2 µm filter prior to dialysis using a cellulose membrane with MWCO 1000 Da. Finally, a yellow brownish solution of luminescent CDs was obtained. The sample of CDs without surface passivation with PEG was named as CDs-X-Y (where X is reaction temperature, and Y is reaction time) while PEG modified CDs was named as CDs-PEG-X-Y accordingly.

### Characterization of CDs

Photoluminescence, PL, property of CDs was analyzed using a spectrofluorometer (JASCO FP-6200, Japan). The emission intensity was measured by varying excitation wavelength between 240 and 400 nm. UV-Vis absorption spectra were recorded at room temperature (25 °C) by UV-Vis spectrophotometer (UV-1800, Shimadzu, Japan). Energy band gap of nanomaterial was determined using Tauc Plot technique^28^. For the measurement of size distribution and zeta potential of synthesized CDs, CDs solution was injected into the folded capillary cell and analyzed for hydrodynamic diameter and surface charges of nanoparticles using Dynamic light scattering (DLS) and Zeta sizer (Zetasizer nano ZS, Malvern Panalytical, UK), respectively. The measurement was carried out three times and the average values with standard deviations were reported.

Morphology and average particle size of CDs and CDs-PEG were analyzed by High-resolution transmission electron microscope (HR-TEM) (JEM-2100 PLUS, Jeol, South Korea) with the accelerating voltage of 200 kV. The CDs solution was dropped onto the TEM grids and dried at low temperature prior to the microscopic analysis. X-ray photoelectron spectroscopic (XPS) analysis was applied to characterize the elemental structure on the surface of CDs samples (Axis ultra DLD, Kratos, UK). Fourier transform infrared spectroscopy (FT-IR) was recorded using Nicolet 6700, Thermo scientific, USA with attenuated total reflectance (ATR) measurement mode to analyze the functional groups of CDs. The spectra were collected between 600 and 4000 cm^−1^ with 100 scans and 4 cm^−1^ resolutions. The Raman spectroscopic analysis was conducted with a laser excitation wavelength at 532 nm (XploRA plus, HORIBA, Japan). The thermal degradation and thermal stability of CDs and CDs-PEG were evaluated by Thermogravimetric analyzer (TG 209 F3 Tarsus, NETZCH, Germany). The sample was heated from 30 to 800 °C at the heating rate of 10 °C/min under nitrogen atmosphere.

The mass yield of unmodified CDs and PEG functionalyzed CDs was calculated from CDs weight (dry basis) in an exact volume of CDs solution after freeze drying (at -80 °C for 48 h) devided by initial precursor weight (dry basis) as shown in Eq. (1). In case of unmodified CDs, the dry weight of substrate was initial weight of EFB, while initial weight of EFB + PEG was used for PEG functionalized CDs.

1 $$Mass\,yield \left( \% \right) = \frac{{Concentration\, of\, CD\, in\, sample\, \left( {g/mL} \right) \times total\, volume\, of\, CDs\, solution\, \left( {mL} \right)}}{Dry\, weight\, substrate\, \left( g \right)} \times 100$$

### DOX loading and release efficiency of CDs-PEG-DOX

For DOX loading, the different ratios of CDs-PEG and DOX solution were investigated. Various volumes of stock solution of DOX and CDs-PEG at 1 mg mL^−1^ were mixed and stirred at 200 rpm at room temperature (25 °C) for 24 h in the dark. Then, 10 mL of mixture at different CDs-PEG to DOX ratios was dialyzed against deionized water (100 mL) in the dark for 2 h to remove unloaded-DOX, and then the CDs-PEG-DOX product was stored at 4 °C. The amount of DOX loaded and released was calculated from Eqs. (2) and (3) when the concentration of DOX was determined using UV absorbance at 480 nm compared with the known concentrations of DOX from the calibration curve. The loading efficiency (DLE) and loading content (DLC) of DOX onto CDs-PEG were calculated using following equations:

2 $$DLE\left( \% \right) = \frac{{\left( {Total\, amount\,of\,DOX - The\, amount\, of\,DOX\, dialyzed\, in\, deionized\, water} \right)}}{Total\, amount\, of\,DOX} \times 100$$

3 $$DLC\left( {mg g^{ - 1} } \right) = \frac{{\left( {Total\, amount\, of\, DOX - The\, amount\, of\, DOX\, dialyzed\, in\, deionized\, water} \right)}}{{Total\, amount\, of\, CDs{\text{-}}PEG}}$$

The effect of pH on DOX release from CDs-PEG-DOX was studied in phosphate buffered saline (PBS) solution at pH 5.0 and 7.4 at 37 °C under 200 rpm stirring speed for 96 h. The amount of DOX released was calculated from UV-Vis absorption at 480 nm compared with the known concentration of DOX from the calibration curve.

### Characterization of CDs-PEG-DOX

The as-prepared CDs-PEG-DOX conjugates were characterized by UV-Vis spectroscopy (UV-1800, Shimadzu, Japan), Fluorescence spectroscopy (JASCO FP-6200, Japan), and zeta potential measurement (Zetasizer nano ZS, Malvern Panalytical, UK). The binding of CDs-PEG with DOX was additionally analyzed by Matrix Assisted Laser Desorption Ionization Time-to-Flight (MALDI/TOF, Autoflex Speed, Bruker, Germany) equipped with a 355 nm Nd:YAG laser in reflectron mode. Each spectrum was the cumulative average of 1000 laser shots at 2000 Hz frequency in a pixel with a diameter of 100 μm. A sampling rate of 2.5 GS s^−1^ was applied to detect ions in the mass range of m/z 0–1000.

### Cell culture and In vitro Cytotoxicity test of CDs-PEG-DOX

Human primary dermal fibroblast normal cells (HDFn), human colorectal adenocarcinoma cells (CaCo-2 cells), and colorectal adenocarcinoma (HT-29) cells were obtained from the American Type Culture Collection (ATCC) (Manassas, Virginia, USA). A mouse fibrosarcoma (L-929) cell line was kindly donated by Associate Professor Dr. Jasadee Kaewsrichan (Drug Delivery System Excellence Center, Faculty of Pharmaceutical Sciences, Prince of Songkla University, Songkhla, Thailand). All cells were cultured in Dulbecco's Modified Eagle Medium (DMEM) supplemented with 10% fetal bovine serum (FBS), 1% penicillin/streptomycin. The cytotoxicity test of CDs-PEG-DOX and CDs-PEG was performed using the WST-1 assay. Briefly, HDFn and fibroblast or CaCo-2 cells in 96 well plates (2 × 10^5^ cells/well) were maintained in DMEM culture medium for 24 h. Then, the old medium was removed, and cells were washed twice with PBS buffer. Cells were treated with the different concentrations of CDs-PEG-DOX or CDs-PEG ranging from 0.7812 to 100% (V/V). The 2% SDS and PBS were used as a positive and negative controls, respectively. The experiment was performed in triplicate. After 24 h, the cell culture media were removed. Cells were washed twice with PBS and re-suspended in fresh DMEM containing 10 μL of WST solution at total volume of 100 μL per well. Cells were incubated for 30 min and the reaction color was determined by measuring optical density at 450 nm using a microplate reader. The measured biological values (optical density: OD) were taken for calculation on the percentage of cell viability using the following mathematical equation:

4 $$\% Cell \,viability = \frac{{Mean\,optical\,density\, \left( {OD} \right)\, of\, cells\, of\, treatment\, group}}{{Mean\, optical\, density\, \left( {OD} \right) \,of\, cells\, of\, control\, group }} \times 100.$$

### Cell uptake and imaging of CDs-PEG and CDs-PEG-DOX

The study of cellular uptake of CDs-PEG and CDs-PEG-DOX in CaCo-2 cells was evaluated. CaCo-2 cells at the concentration of 1 × 10^5^ cells per well in DMEM supplemented with 10% FBS and 1% penicillin/streptomycin were seeded in a 24-well plate for the assays. The cells were allowed to attach to the slip overnight before switching to serum-free media. After that, Free DOX, CDs-PEG, CDs-PEG-DOX, and PBS at their original concentrations were added and incubated for 1 h and 5 h. The cell cultures were then washed with PBS to remove any non-binding sample. For the cell fixation, 4%wt Paraformaldehyde (PFA) was added into the cell solution and incubated for 30 min before washing three times with PBS. The nuclei of fixed cells were stained with Honest (1:1000, 10 mg mL^−1^ in PBS), left for 20 min in the dark, and then washed twice with PBS. Afterward, the samples from the wells were dropped onto the glass slide and covered with the cover slips. Excess liquid was removed with filter paper. After 15 min in the dark, the cover slip was sealed with nail polishing solution. The fixed cells were stored at 4 °C in the dark. Finally, fluorescent microscopy (Olympus IX71, Japan) and stereomicroscopy (Olympus SZX16, Japan) were used to capture cell images.

## Supplementary Information

Supplementary Information.

## Data Availability

All data generated or analyzed during this study are included in this published article and its supplementary information files.
