# Supplementary material for: Facile preparation of aqueous-soluble fluorescent polyethylene glycol functionalized carbon dots from palm waste by one-pot hydrothermal carbonization for colon cancer nanotheranostics
Source: Sci Rep. 2022 Jun 22;12:10550. doi: 10.1038/s41598-022-14704-x (PMC9217983; doi:10.1038/s41598-022-14704-x)
Supplement: Supplementary file 1 — Supplementary Information. [file 41598_2022_14704_MOESM1_ESM.pdf]

Supporting Information

Facile preparation of aqueous-soluble fluorescent polyethylene glycol functionalized carbon dots from palm waste by one-pot hydrothermal carbonization for colon cancer nanotheranostics

Amornrat Sangjan[a], Suthida Boonsith[a], Kanokwan Sansanaphongpricha[b,*], Tapanee Thinbanmai[b], Sakhon Ratchahat[a], Navadol Laosiripojana[c], Kevin C.-W. Wu,[d-f] Hyeon Suk Shin[g-j] Chularat Sakdaronnarong[a,**]

[a] Department of Chemical Engineering, Faculty of Engineering, Mahidol University, 999 Putthamonthon 4 Road, Salaya, Putthamonthon, Nakorn Pathom 73170, Thailand.

[b] National Nanotechnology Center (NANOTEC), National Science and Technology Development Agency (NSTDA), 111 Thailand Science Park, Phahonyothin Road,

Khlong Nueng, Khlong Luang, Pathum Thani 12120, Thailand.

c The Joint Graduate School of Energy and Environment (JGSEE), King Mongkut's University of Technology Thonburi, 126 Pracha Uthit Road, Bang Mot, Tungkru, Bangkok 10140, Thailand.

[d] Department of Chemical Engineering, National Taiwan University, No.1, Sec.4 Roosevelt Road, Taipei, 10617 Taiwan.

[e] Center of Atomic Initiative for New Materials (AI-MAT), National Taiwan University, Taipei 10617, Taiwan.

[f] International Graduate Program of Molecular Science and Technology, National Taiwan University (NTU-MST), Taipei 10617, Taiwan.

[g] Department of Energy Engineering, Ulsan National Institute of Science & Technology (UNIST), Ulsan 44919, Republic of Korea.

[h] Department of Chemistry, UNIST, Ulsan 44919, Republic of Korea.

[i] Center for Multidimensional Carbon Materials, Institute of Basic Science (IBS), Ulsan 44919, Republic of Korea.

[j] Low Dimensional Carbon Material Center, UNIST, Ulsan 44919, Republic of Korea

*Corresponding Author's E-mail: kanokwan.san@nanotec.or.th

**Corresponding Author's E-mail: chularat.sak@mahidol.ac.th

Table S1. Summary of CDs and CDs-PEG properties from hydrothermal synthesis.

Sample	Fluorescence Property			Physical property		
	Excitation wavelength (nm)	Max. Emission wavelength (nm)	PL Intensity (a.u.)	Zeta potential (mV)	Hydrodynamic size (nm)	Mass yield (%)
CDs-180C-6h	340	420	107.89	-7.77	579.1	3.01
CDs-220C-6h	350	429	135.05	-24.23	412.90	1.20
CDs-PEG	240	403	397.74	-36.5	2,434.33	33.73

Table S2. Distribution of functional groups based on the deconvolution model of XPS C 1s and O 1s peaks of CDs-220C-6h and CDs-220C-10h.

Material	Relative content of each component peak								Sp^2/Sp^3
	C 1s (Atomic %)				O 1s (Atomic %)				
	C_1[a]	Sp^2[b]	Sp^3[c]	C_2[d]	O_1[e]	O_2[f]	O_3[i]	O_4[j]	
CDs-220C-6h	-	46.75	45.53	7.72	-	-	86.36	13.64	1.03
CDs-220C-10h	-	54.78	37.68	7.54	-	-	74.29	25.71	1.45
CDs-PEG	79.7	-	20.3	-	86.36	13.64	-	-	-

Note [a] The peak at 283.1 eV represents the π-bonded carbon atom, [b] the peak at 284.5 eV represents the sp^2-bonded carbon atom, [c] the peak at 285.6 eV represents the sp^3-bonded carbon atom, [d] the peak at 288 eV represents the C-O-C component, [e] the peak at 530.5 eV represents the C=O component, [f] the peak at 531 eV represents the C-OH and C-O-C component, [i] the peak at 531.6-531.9 eV represents the C=O component, and [j] the peak at 532.8 eV represents the C-OH and C-O-C component.

Table S3. Elemental composition of CDs-220C-6h and CDs-220C-10h measured from XPS analysis.

Material	Atomic concentration (%)				Mass concentration (%)				Atomic ratio		
	C 1s	O 1s	N 1s	S $2p^2$	C 1s	O 1s	N 1s	S $2p^2$	O/C	N/C	S/C
CDs-220C-6h	64.08	33.24	0.92	1.76	56.15	38.80	0.94	4.11	0.52	0.01	0.03
CDs-220C-10h	68.03	28.95	1.96	1.06	60.90	34.53	2.05	2.52	0.43	0.03	0.02
CDs-PEG	72.53	27.25	0.00	0.06	66.44	33.49	0.00	0.06	0.38	0.00	0.00

Table S4. Proximate and ultimate compositional analysis of EFB.

Hemicellulose (% wt)	Cellulose (% wt)	Lignin (% wt)	Ash (% wt)	HHV (MJ kg^{-1})	Elemental content (%wt)					Atomic ratio		
					C	H	N	O	S	O/C	N/C	S/C
19.78	49.02	18.61	1.85	13.02	42.25	5.88	1.03	50.75	0.09	0.900	0.021	0.001

Table S5. Investigation ratio of CDs-PEG and DOX to value of maximum loading efficiency (DLE) and maximum loading content (DLC) of DOX

Conditions	CDs-PEG in solution (mg mL^{-1})	DOX in solution (μg mL^{-1})	DLE (%)	DLC (mg g^{-1})
1	1	50	48.61	97.21
2	1	100	94.61	94.61
3	1	200	179.49	89.42

Table S6. The cell cytotoxicity test data of CDs-PEG-DOX and CDs-DOX toward CaCo-2 and Fibroblast cells

Concentration ($\mu g\ mL^{-1}$)	%Cell viability			
	Fibroblast	SD	CaCo-2	SD
		CDs-PEG		
4000	27.42	0.009	37.17	0.005
2000	72.94	0.077	59.75	0.006
500	82.77	0.006	60.13	0.004
62.50	84.05	0.017	76.6	0.004
3.91	92.76	0.009	80.57	0.002
0.12	95.51	0.01	85.97	0.016
0.002	98.73	0.009	92.89	0.004
0.00001	99.9	0.002	98.43	0.003
Control	100	0	100	0
		CDs-PEG-DOX		
2937.72	28.24	0.013	33.9	0.003
1468.86	81.86	0.011	67.86	0.015
367.21	86.03	0.002	77.36	0.004
45.90	93.58	0.027	83.46	0.043
2.87	95.01	0.034	83.58	0.01
0.09	98.01	0.001	84.21	0.035
0.001	99.08	0.004	84.91	0.052
0.00001	108.15	0.003	85.72	0.063
Control	100	0	100	0

Table S7. IC_{50} of CDs, CDs-PEG and CDs-PEG-DOX with different cell lines.

Sample	IC_{50} (μg mL^{-1})	
	L929	HT-29
CDs	637.00	1177.76
CDs-PEG	Not inhibited*	Not inhibited*
CDs-PEG-DOX	35.81	Not inhibited*

*Not inhibited = The sample did not inhibit cell growth at the tested concentration: CDs at 625, 1250, 2500, 5000 and 10,000 μg mL^{-1}, CDs-PEG at 312.5, 625, 1250, 2500, 5000 μg mL^{-1} and CDs-PEG-DOX at 31.25, 62.5, 125, 250, and 500 μg mL^{-1}.

Table S8. The cell cytotoxicity test data of CDs, CDs-PEG and CDs-PEG-DOX toward L929 and HT-29 cells

Concentration (μg mL^{-1})	%Cell viability L929	SD	HT-29	SD
CDs				
0	100.00	0.00	100.00	0.00
625	85.17	4.26	78.04	2.34
1250	15.68	5.48	33.16	0.99
2500	9.74	2.29	5.98	0.18
5000	8.46	1.07	5.82	0.17
10000	5.04	1.22	5.03	0.15
CDs-PEG				
0	100.00	0.00	100.00	0.00
312.5	107.60	0.00	95.48	3.00
625	96.34	0.19	85.27	2.86
1250	88.33	0.38	78.68	2.56
2500	66.61	3.46	77.68	2.36
5000	64.44	0.78	76.6	2.33
CDs-PEG-DOX				
0	100.00	0.00	100.00	0.00
31.25	65.58	3.36	131.34	3.94
62.5	24.49	0.43	95.11	2.85
125	21.48	0.47	73.18	2.20
250	10.03	3.93	55.63	1.67
500	7.45	3.46	55.13	1.65

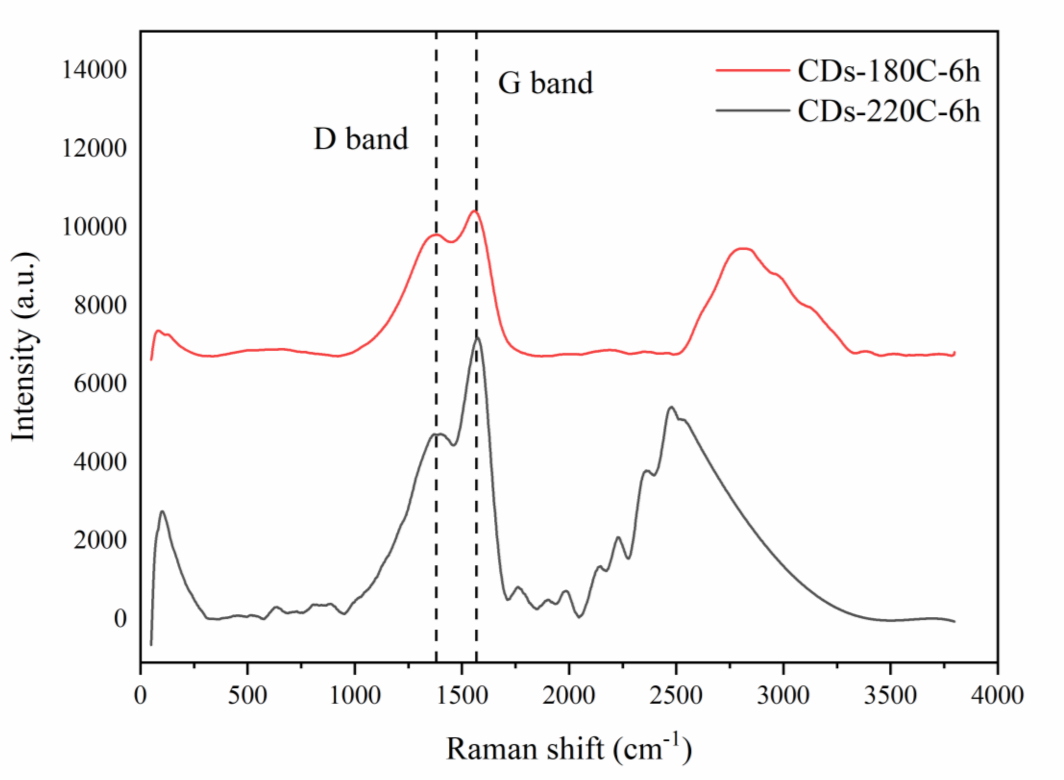

Figure S1. Raman spectra of the CDs-180C-6h and CDs-220C-6h.

	D-spacing	FFT	iFFT	Profile of iFFT
(A) CDs-220C-6h	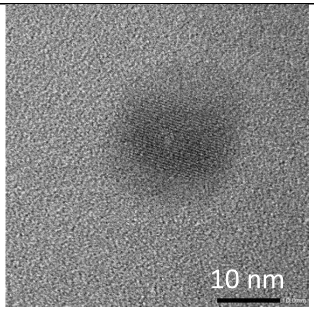	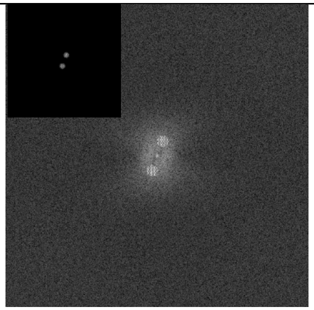	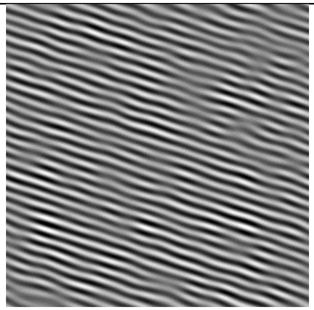	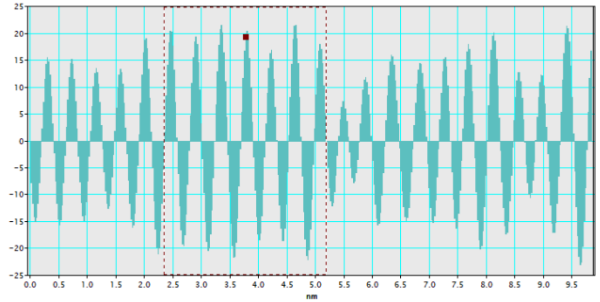
(B) CDs-220C-10h	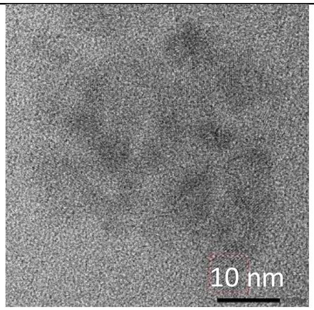	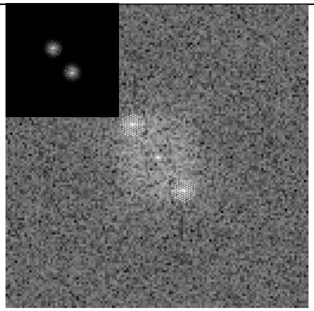	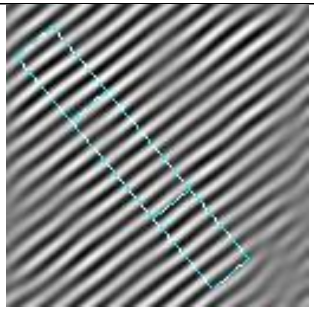	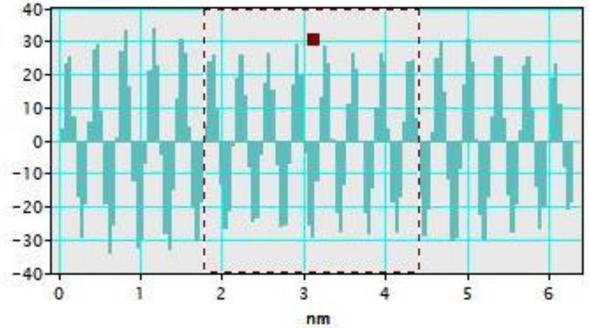
(C) CDs-PEG	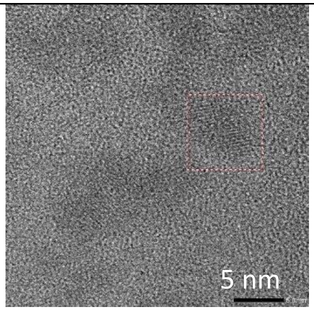	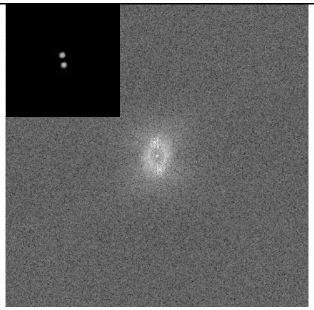	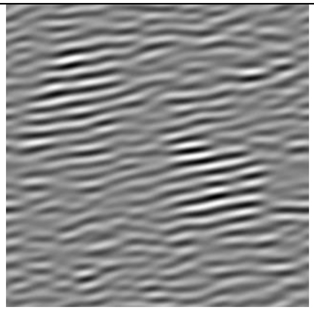	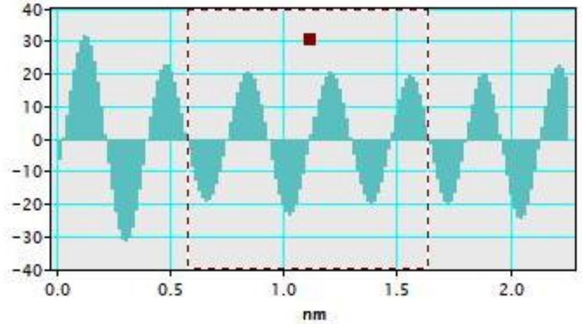

Figure S2. The FFT image of (A) CDs-220C-6h, (B) CDs-220C-10h, and (C) CDs-PEG

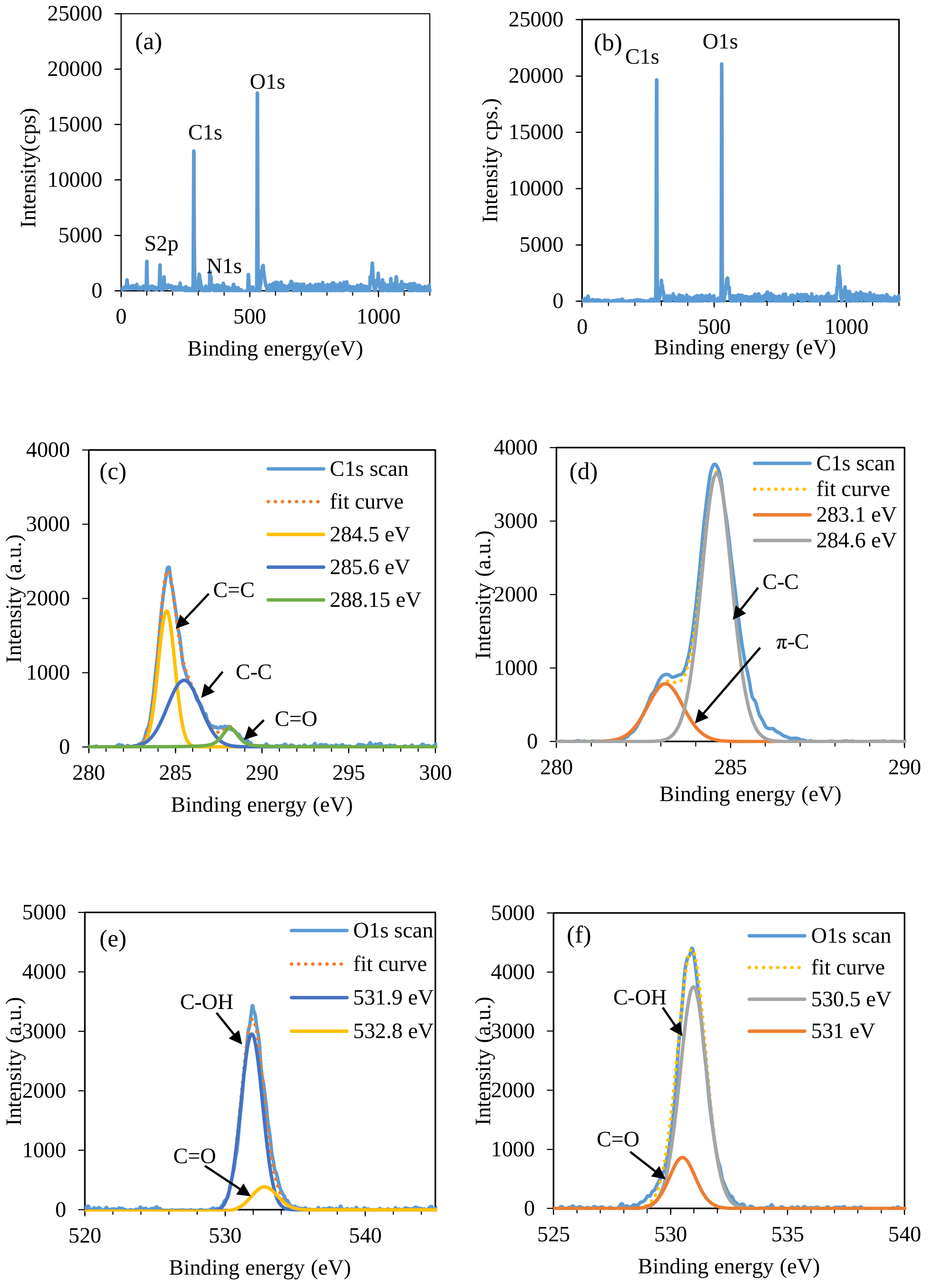

Figure S3. XPS spectra survey of (a) CDs-220C-6h, (b) CDs-PEG, C1s scan of (c) CDs-220C-6h, (d) CDs-PEG, O1s scan of (e) CDs-220C-6h, and (f) CDs-PEG.

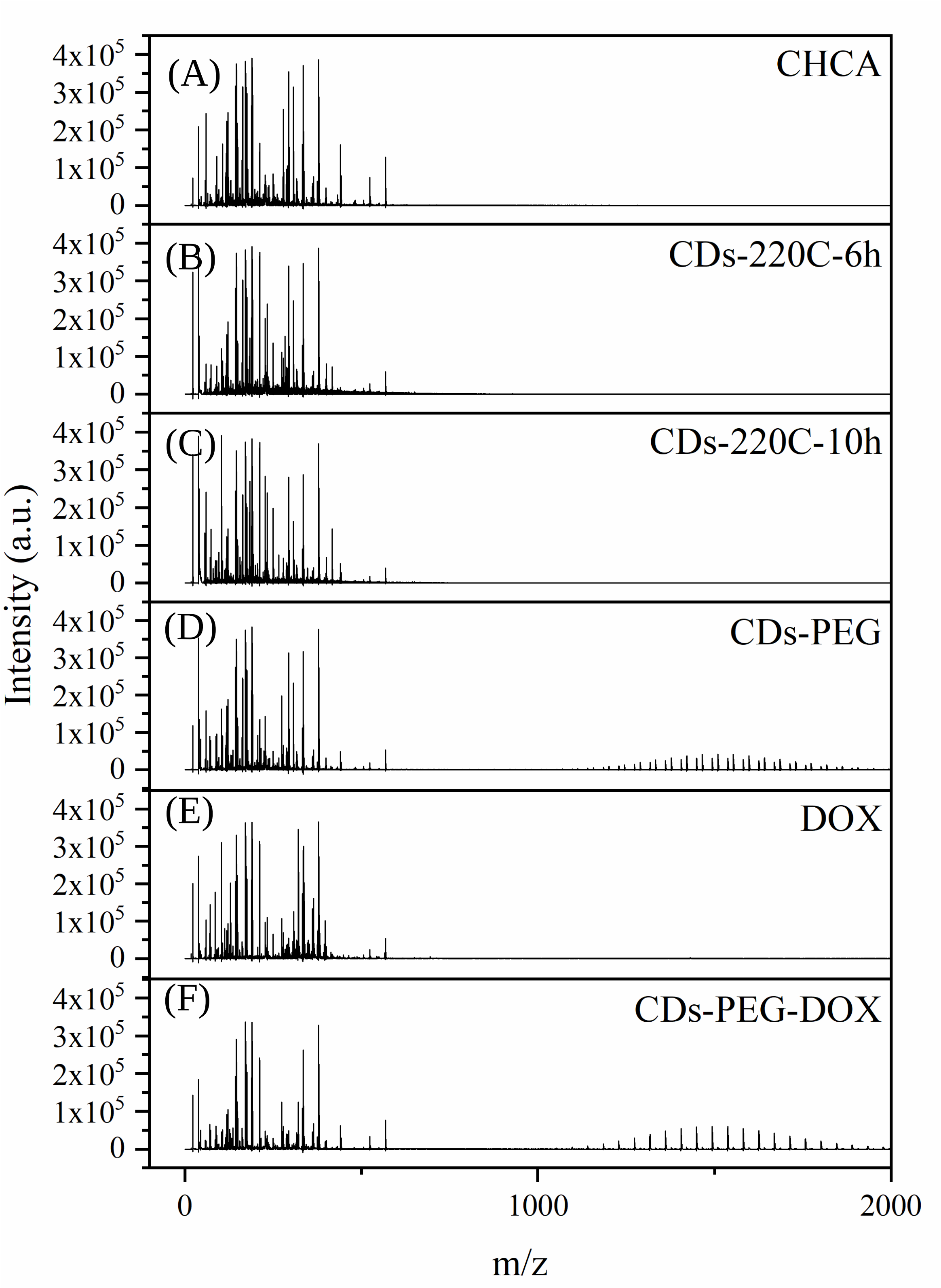

Intensity (a.u.)
4x10^5
3x10^5
2x10^5
1x10^5
0
(A)
CHCA
(B)
CDs-220C-6h
(C)
CDs-220C-10h
(D)
CDs-PEG
(E)
DOX
(F)
CDs-PEG-DOX
0
1000
2000
m/z

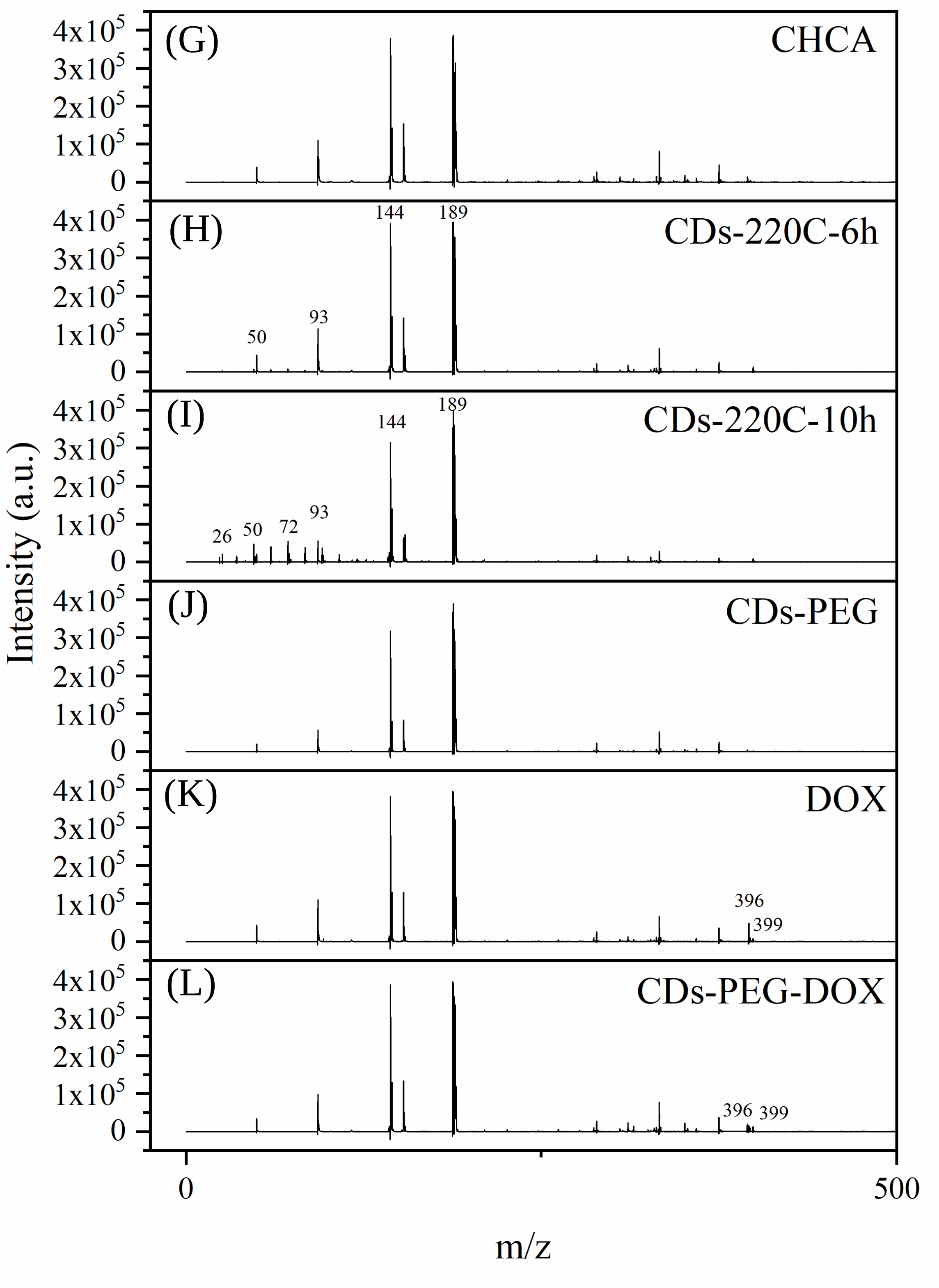

Figure S4. MALDI-TOF-MS spectra of CHCA, CDs-220C-6h, CDs-220C-10h, CDs-PEG, DOX, and CDs-PEG-DOX in positive-ion mode (A-F) and negative-ion mode (G-L).

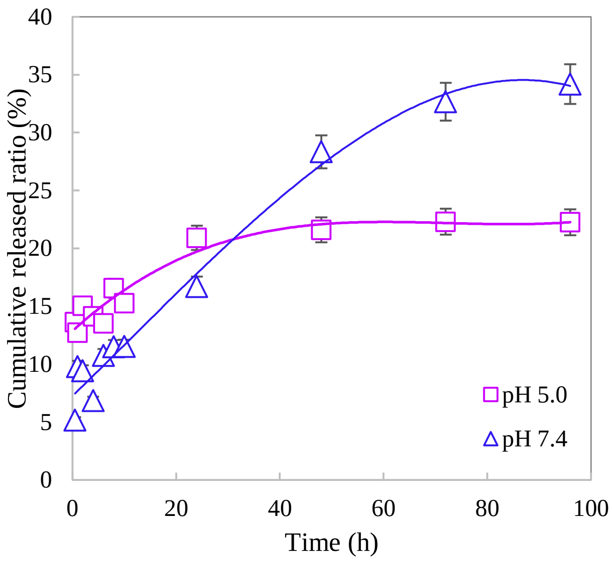

Figure S5. The release of DOX from CDs-PEG-DOX versus time at pH 5.0 and 7.4 under 200 rpm stirring speed at 37°C.

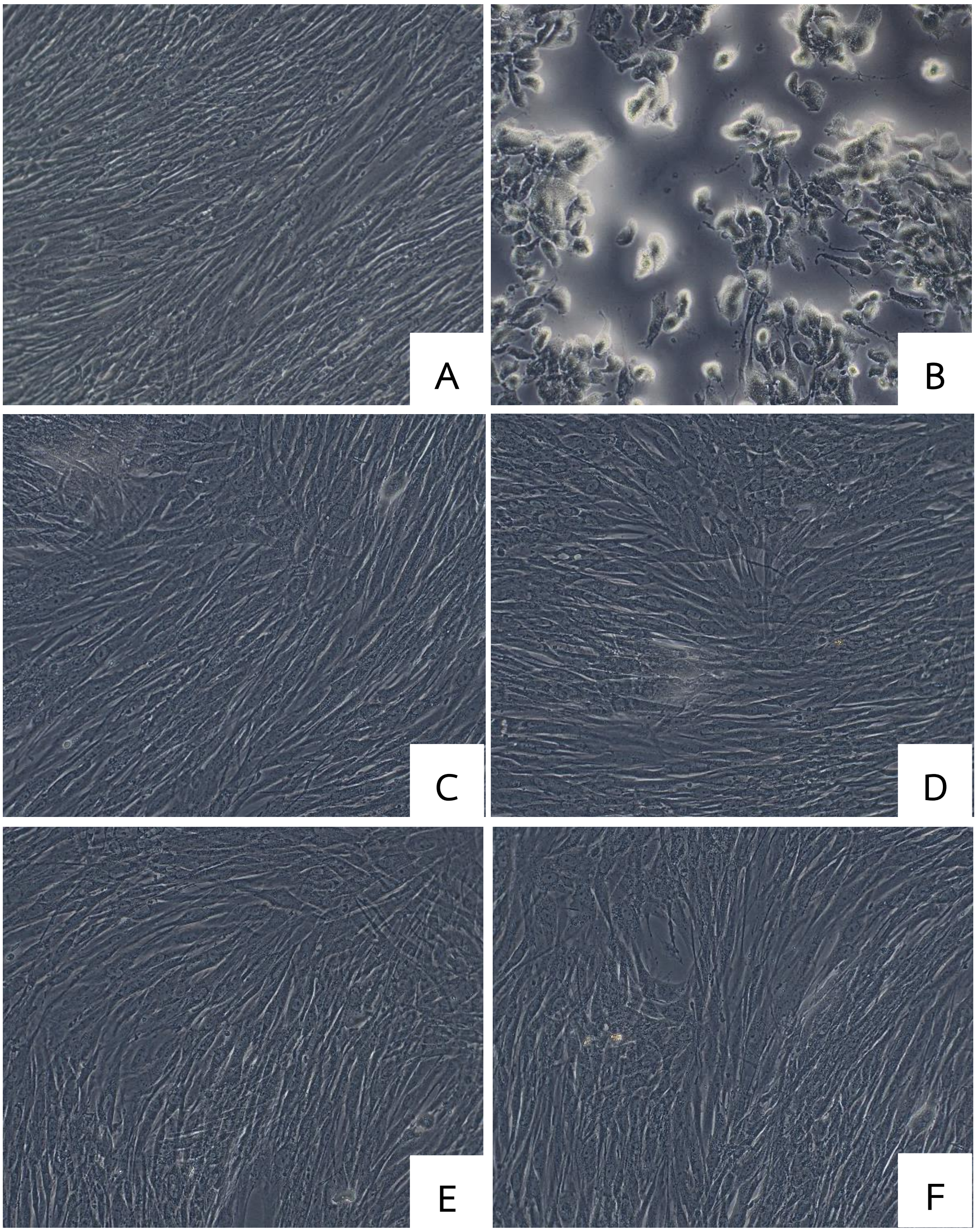

Figure S6. Results of microscopic evaluation of primary fibroblast cells: [A] untreated cells (negative control), [B] 20 µg/ml MMC (positive control), [C] CDs-PEG at 0.78125%, [D] at 1.5625%, [E] at 3.125% and [F] at 6.25%, examined under the inverted microscope (40× magnification)

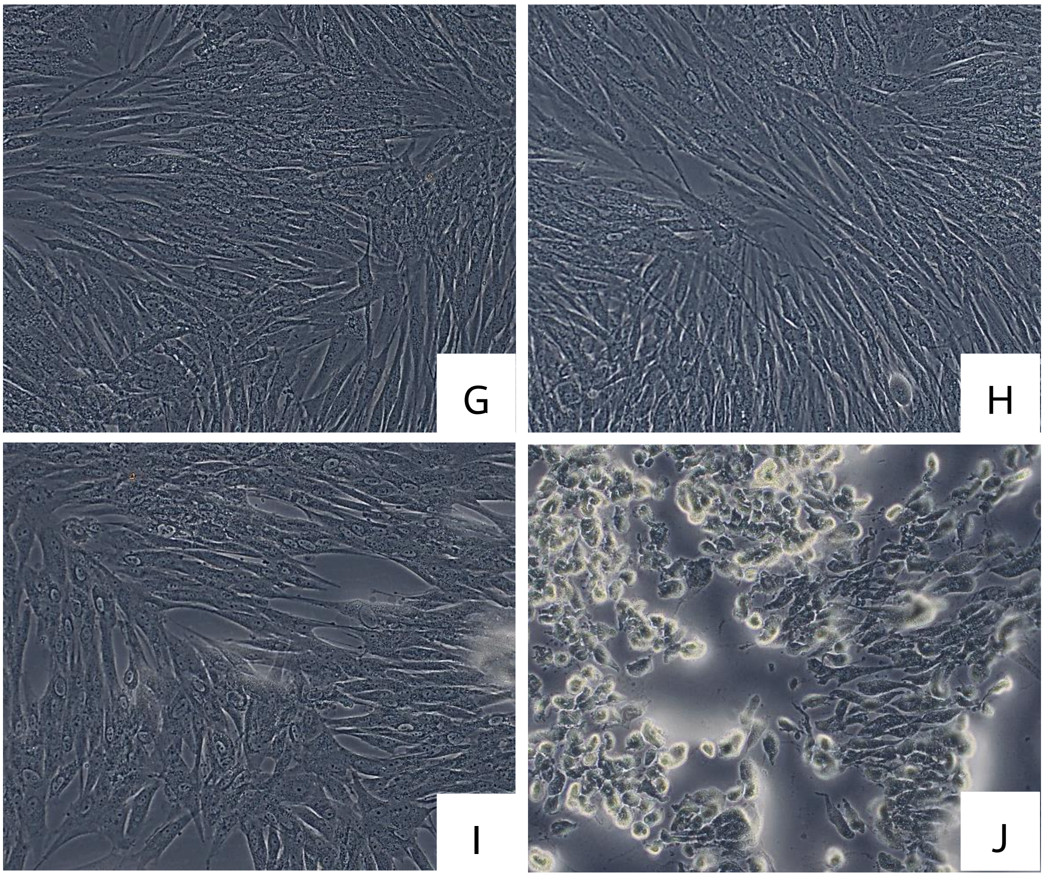

Figure S6 (cont). Results of microscopic evaluation of primary fibroblast cells treated with CDs-PEG; [G] at 12.5%, [H] at 25%, [I] at 50% and [J] at 100% examined under the inverted microscope (40× magnification).

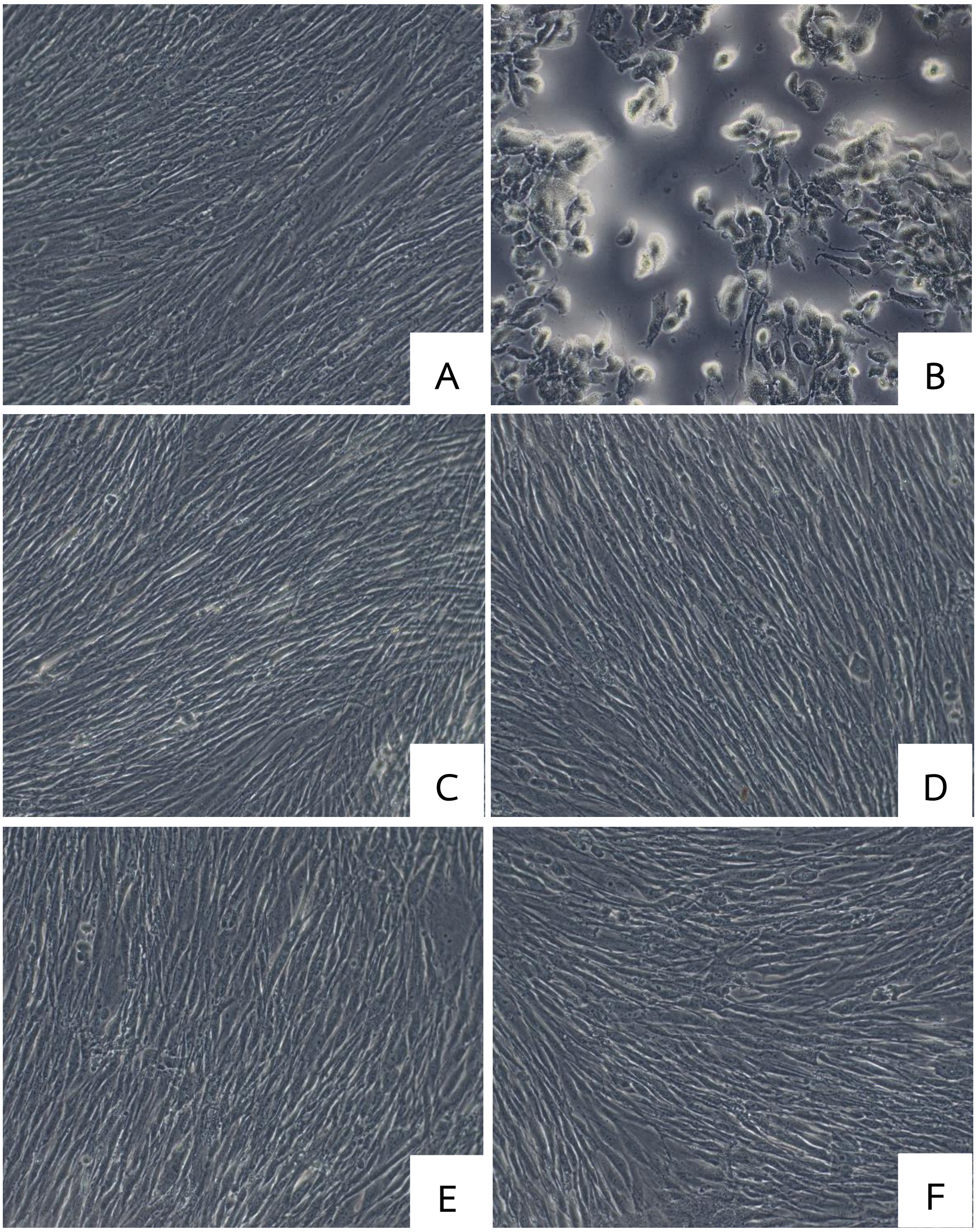

Figure S7. Results of microscopic evaluation of primary fibroblast cells: [A] untreated cells (negative control), [B] 20 µg/ml MMC (positive control), [C] CDs-PEG-DOX at 0.78125%, [D] at 1.5625%, [E] at 3.125% and [F] at 6.25%, examined under the inverted microscope (40× magnification)

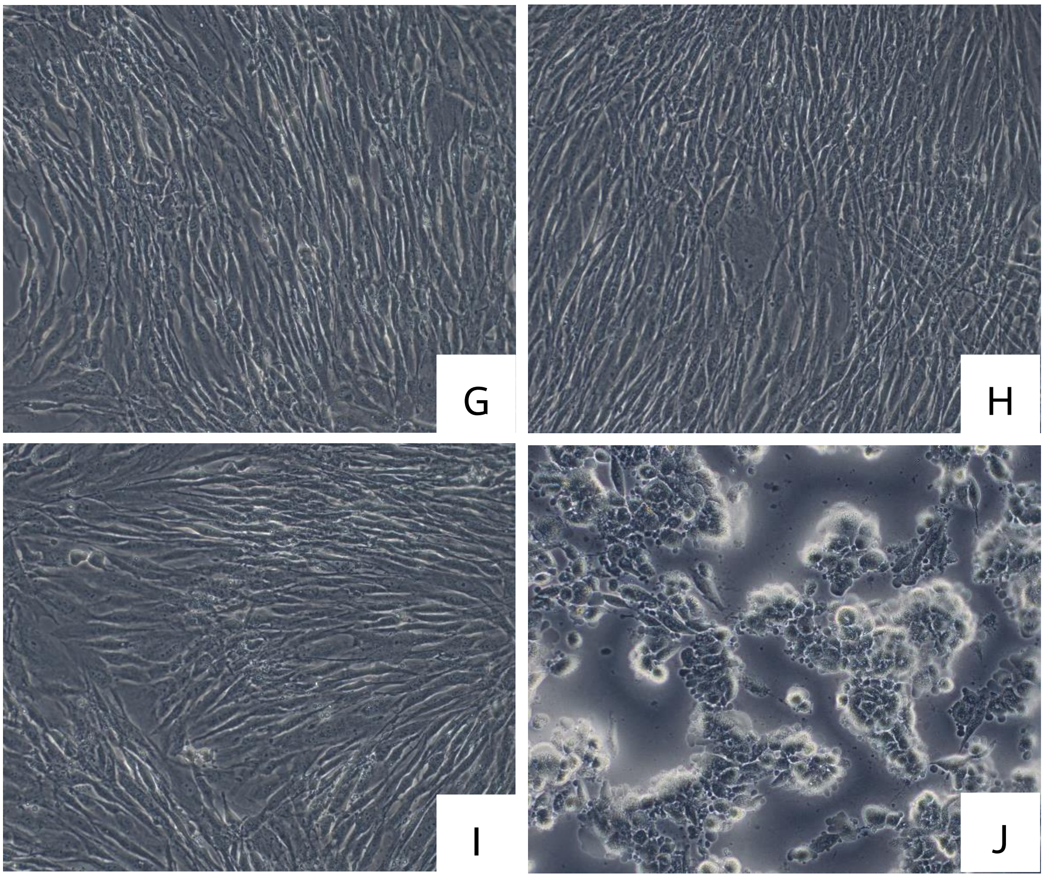

Figure S7. (cont). Results of microscopic evaluation of primary fibroblast cells treated with CDs-PEG-DOX; [G] at 12.5%, [H] at 25%, [I] at 50% and [J] at 100% examined under the inverted microscope (40× magnification).

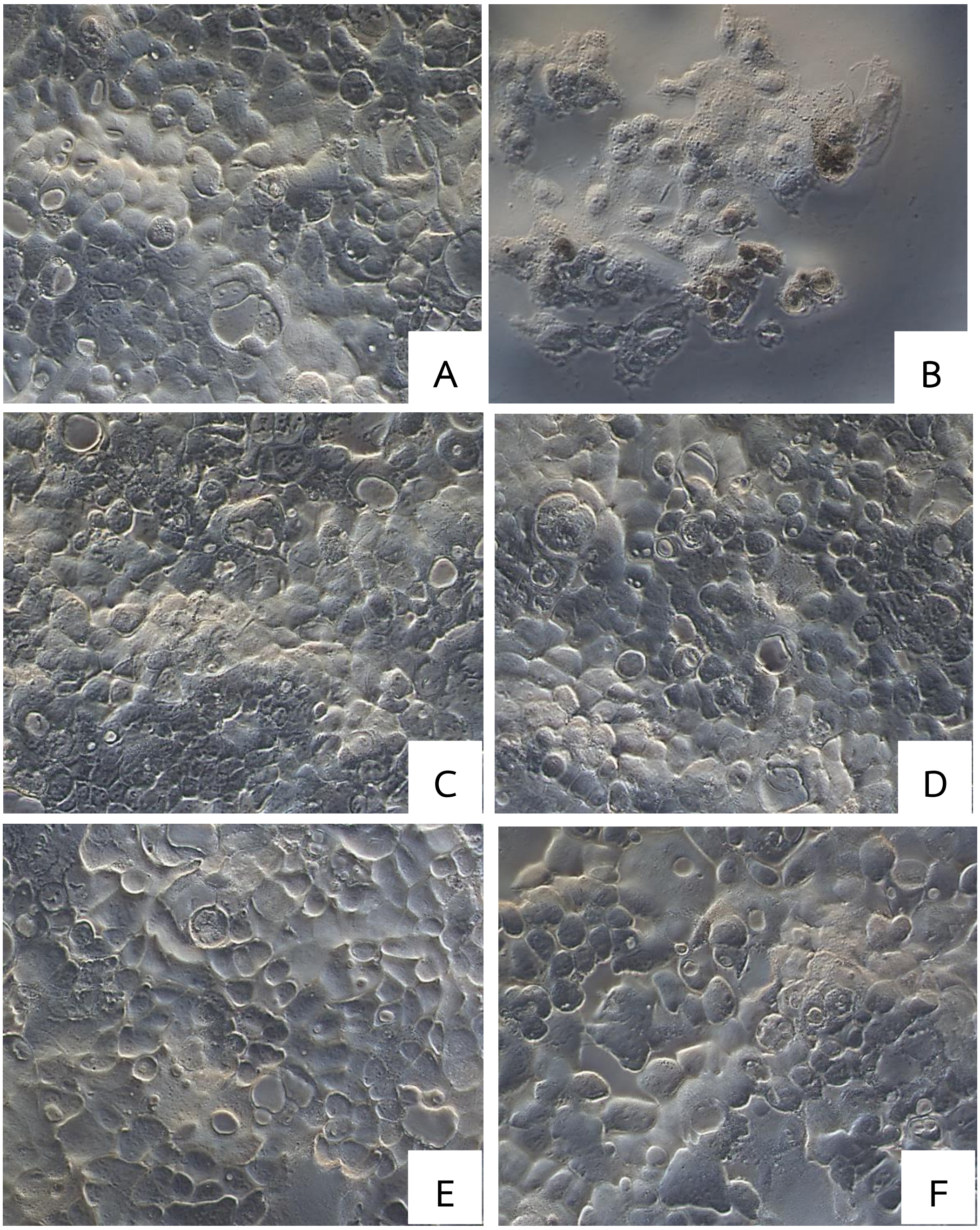

Figure S8. Results of microscopic evaluation of CaCo-2 cells: [A] untreated cells (negative control), [B] 20 µg/ml MMC (positive control), [C] CDs-PEG at 0.78125%, [D] at 1.5625%, [E] at 3.125% and [F] at 6.25%, examined under the inverted microscope (40× magnification)

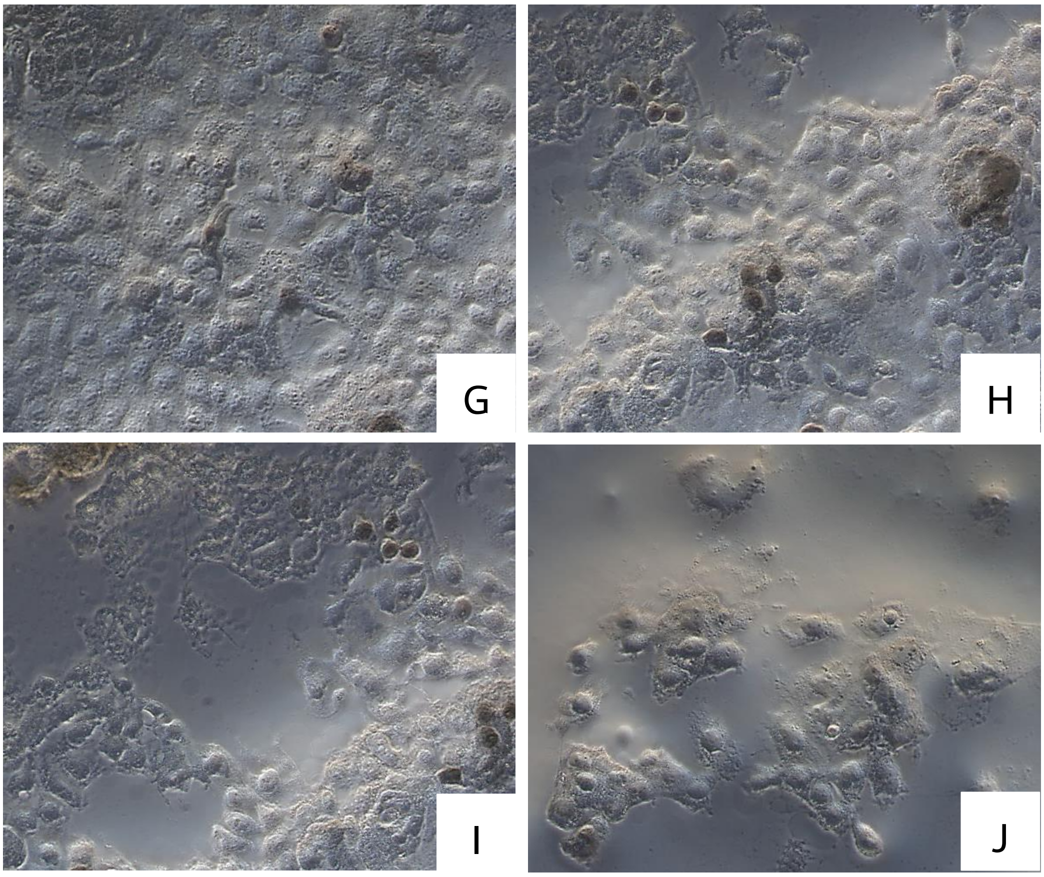

Figure S8 (cont). Results of microscopic evaluation of CaCo-2 cells treated with CDs-PEG; [G] at 12.5%, [H] at 25%, [I] at 50% and [J] at 100% examined under the inverted microscope (40× magnification).

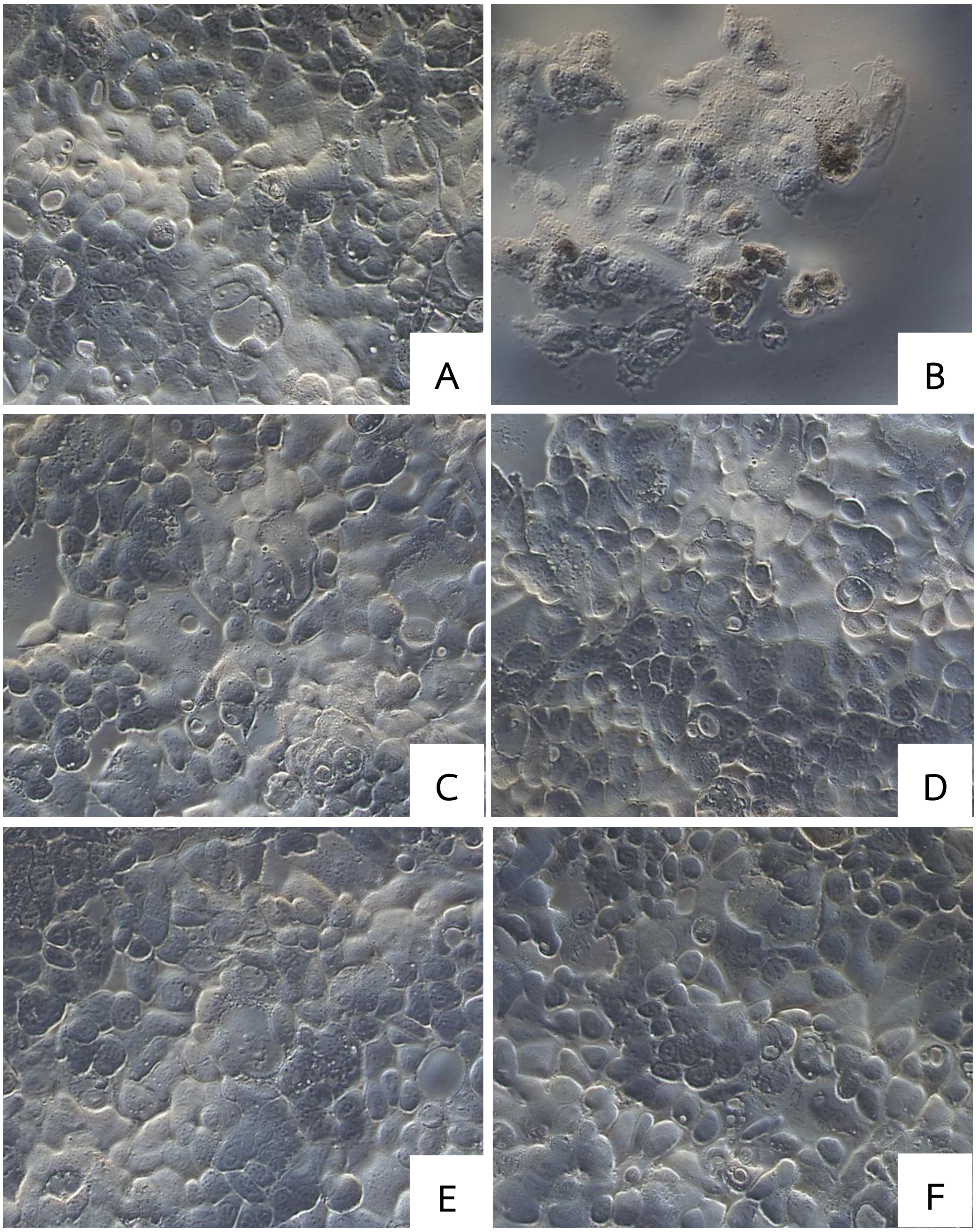

Figure S9. Results of microscopic evaluation of CaCo-2 cells: [A] untreated cells (negative control), [B] 20 µg/ml MMC (positive control), [C] CDs-PEG-DOX at 0.78125%, [D] at 1.5625%, [E] at 3.125% and [F] at 6.25%, examined under 9the inverted microscope (40× magnification)

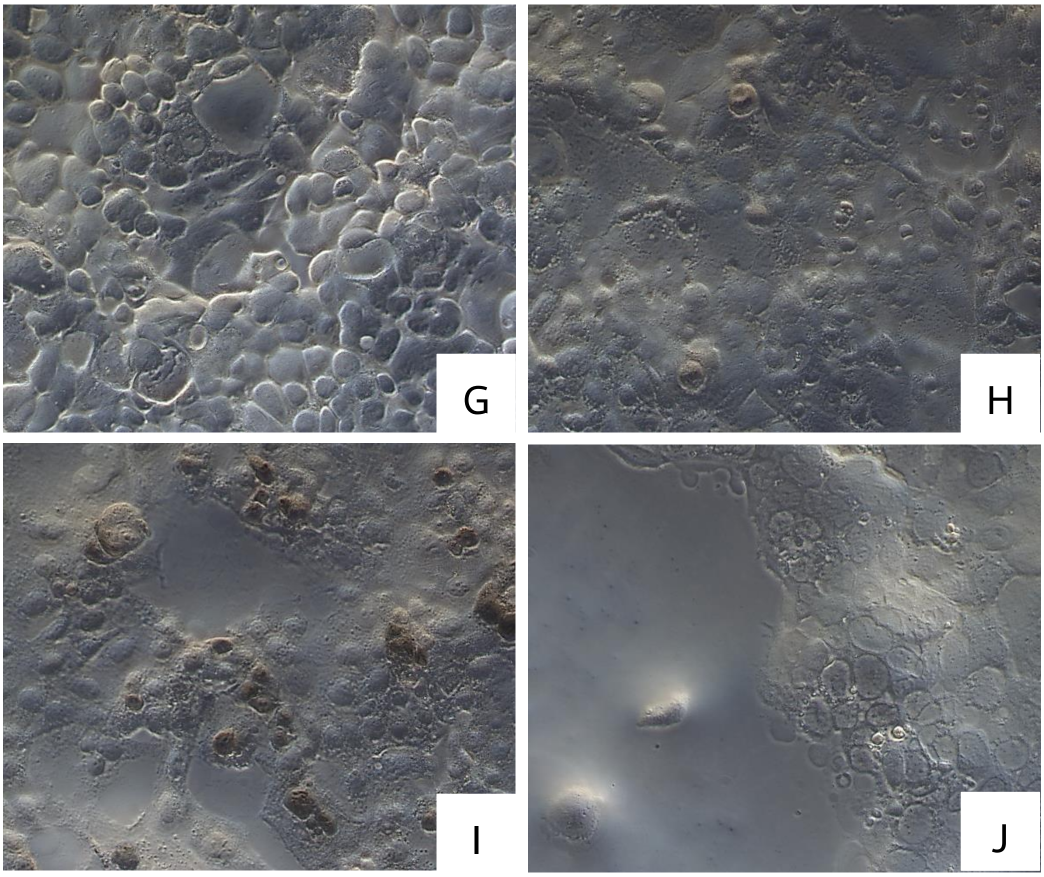

Figure S9. (cont). Results of microscopic evaluation of CaCo-2 cells treated with CDs-PEG-DOX; [G] at 12.5%, [H] at 25%, [I] at 50% and [J] at 100% examined under the inverted microscope (40× magnification).

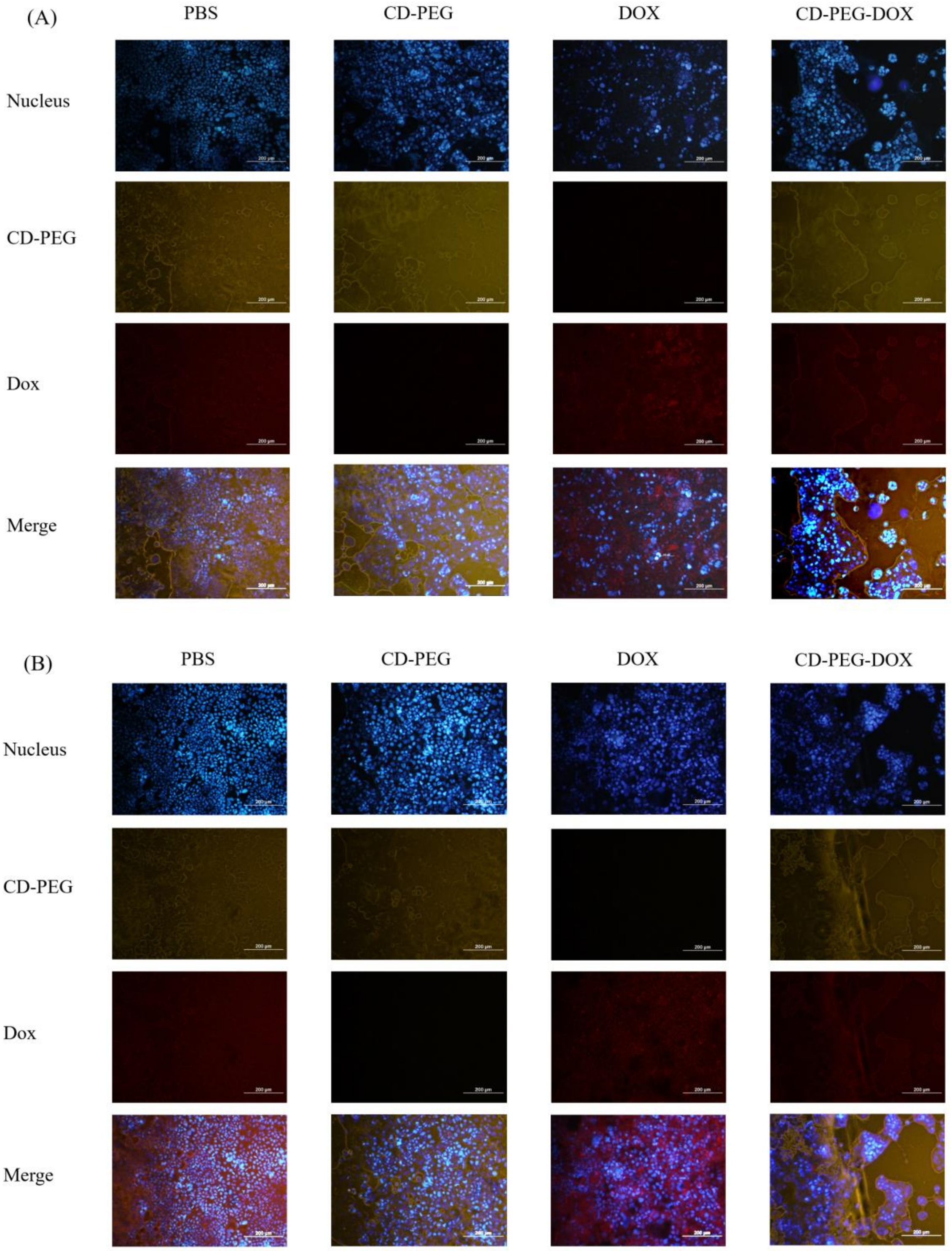

Figure S10. Fluorescence microscopic images of CaCo-2 cells after incubation in PBS, CDs-PEG, DOX, and CDs-PEG-DOX synthesized from 3 mg mL^{-1} of CDs-PEG containing 300 μg mL^{-1} DOX for (A) 1 hour, and (B) 5 hours indicating the translocation of DOX into the cell nuclei from the cytoplasm. (GREEN - CDs-PEG, BLUE - cell nuclei, RED - Doxorubicin).

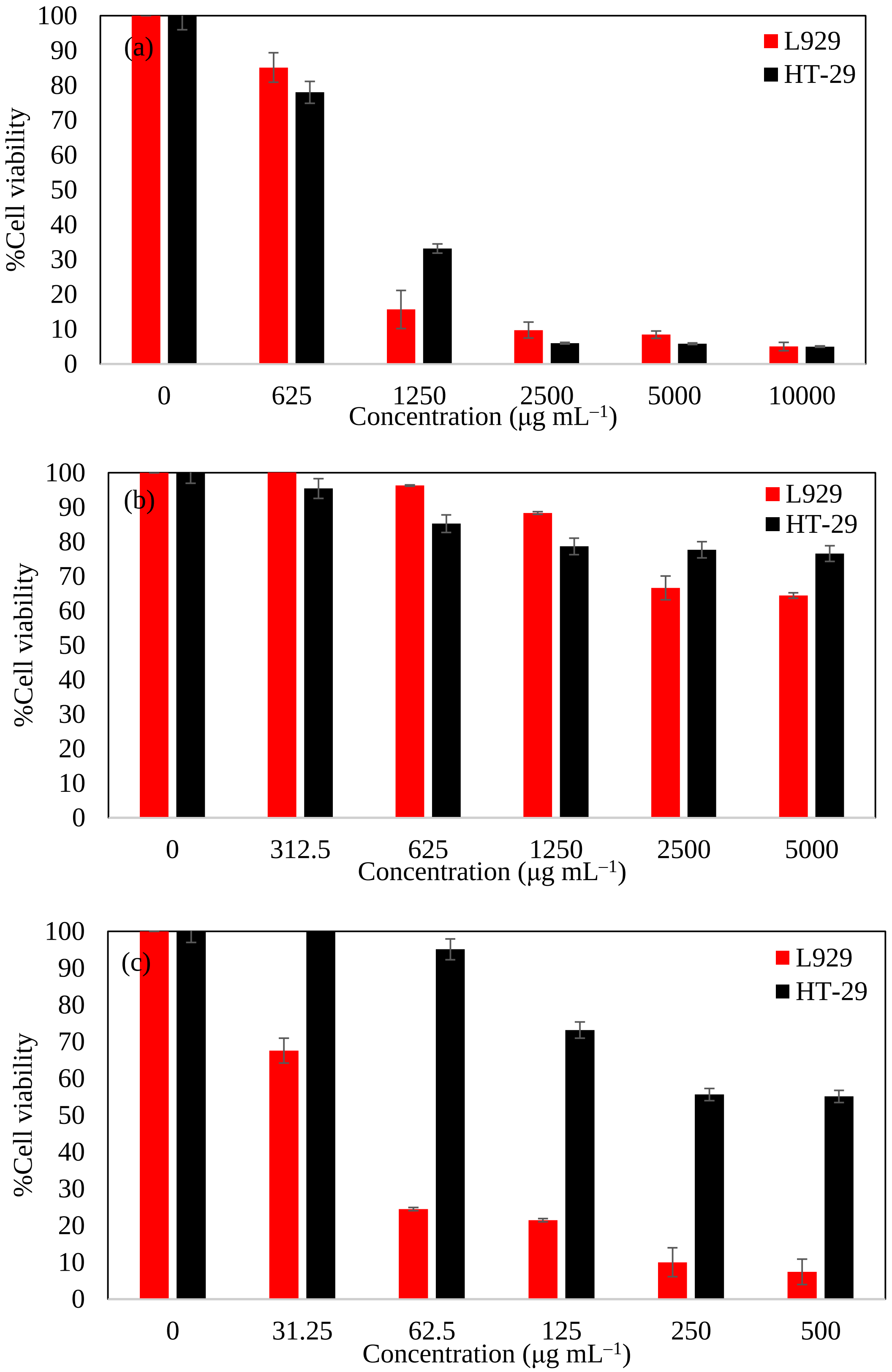

Figure S11. Cytotoxicity with MTT assay of mouse fibroblast cells (L929) and human colorectal adenocarcinoma cells (HT-29) for 72h incubation at different concentrations of (a) CDs from 625-10000 µg mL^{-1}, (b) CDs-PEG from 312.5-5000 µg mL^{-1}, and (c) CDs-PEG-DOX from 31.25-500 µg mL^{-1}.

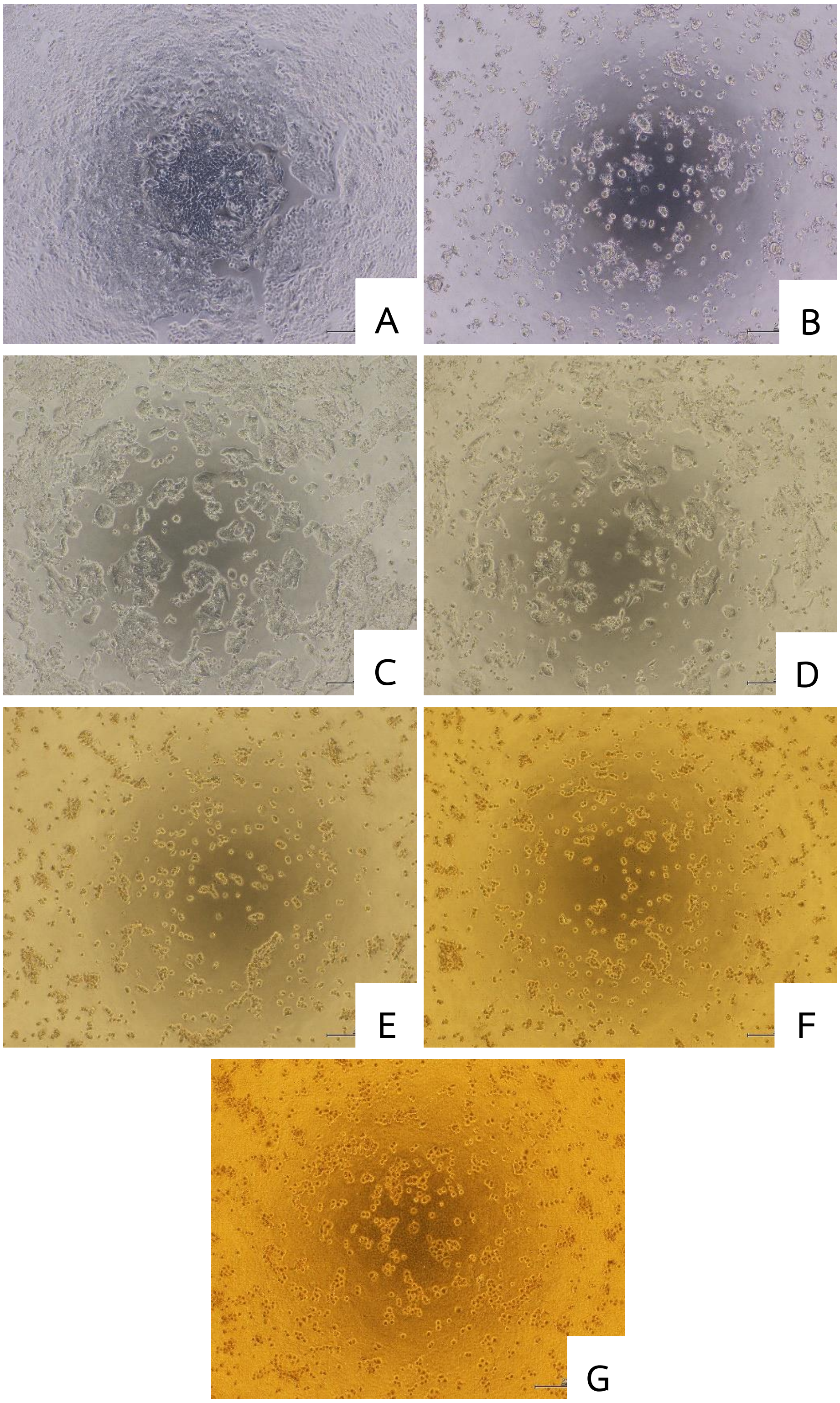

Figure S12. Results of microscopic evaluation of HT-29 cell: [A] untreated cells (negative control), [B] 10 μg mL^{-1} doxorubicin (positive control), [C] CDs at 625 μg mL^{-1}, [D] at 1250 μg mL^{-1}, [E] at 2500 μg mL^{-1}, [F] at 5000 μg mL^{-1}, and [G] at 10000 μg mL^{-1} examined under the inverted microscope (10× magnification)

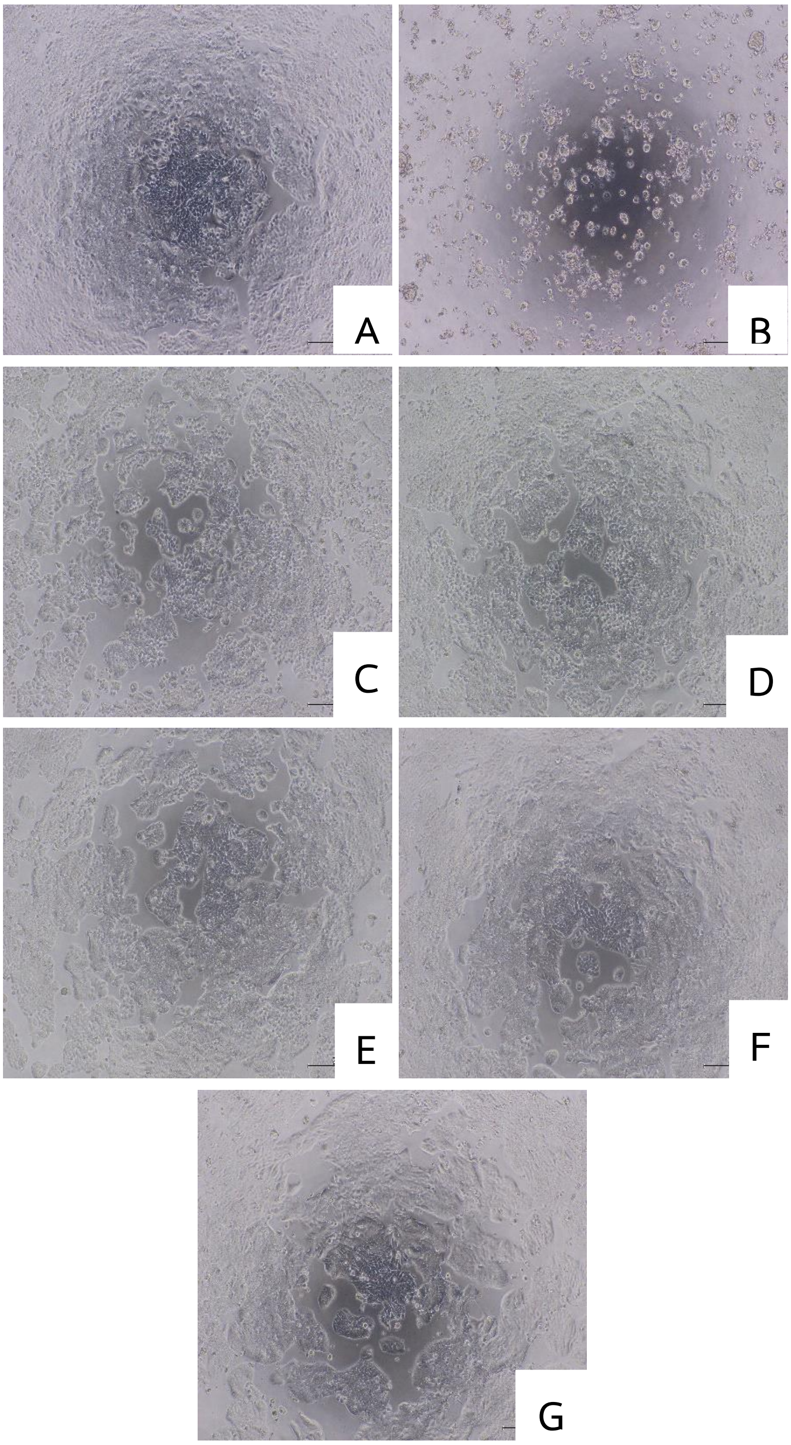

Figure S13. Results of microscopic evaluation of HT-29 cell: [A] untreated cells (negative control), [B] 10 µg mL^{-1} doxorubicin (positive control), [C] CDs-PEG at 312.5 µg mL^{-1} [D] at 625 µg mL^{-1}, [E] at 1250 µg mL^{-1}, [F] at 2500 µg mL^{-1}, and [G] at 5000 µg mL^{-1} examined under the inverted microscope (10× magnification)

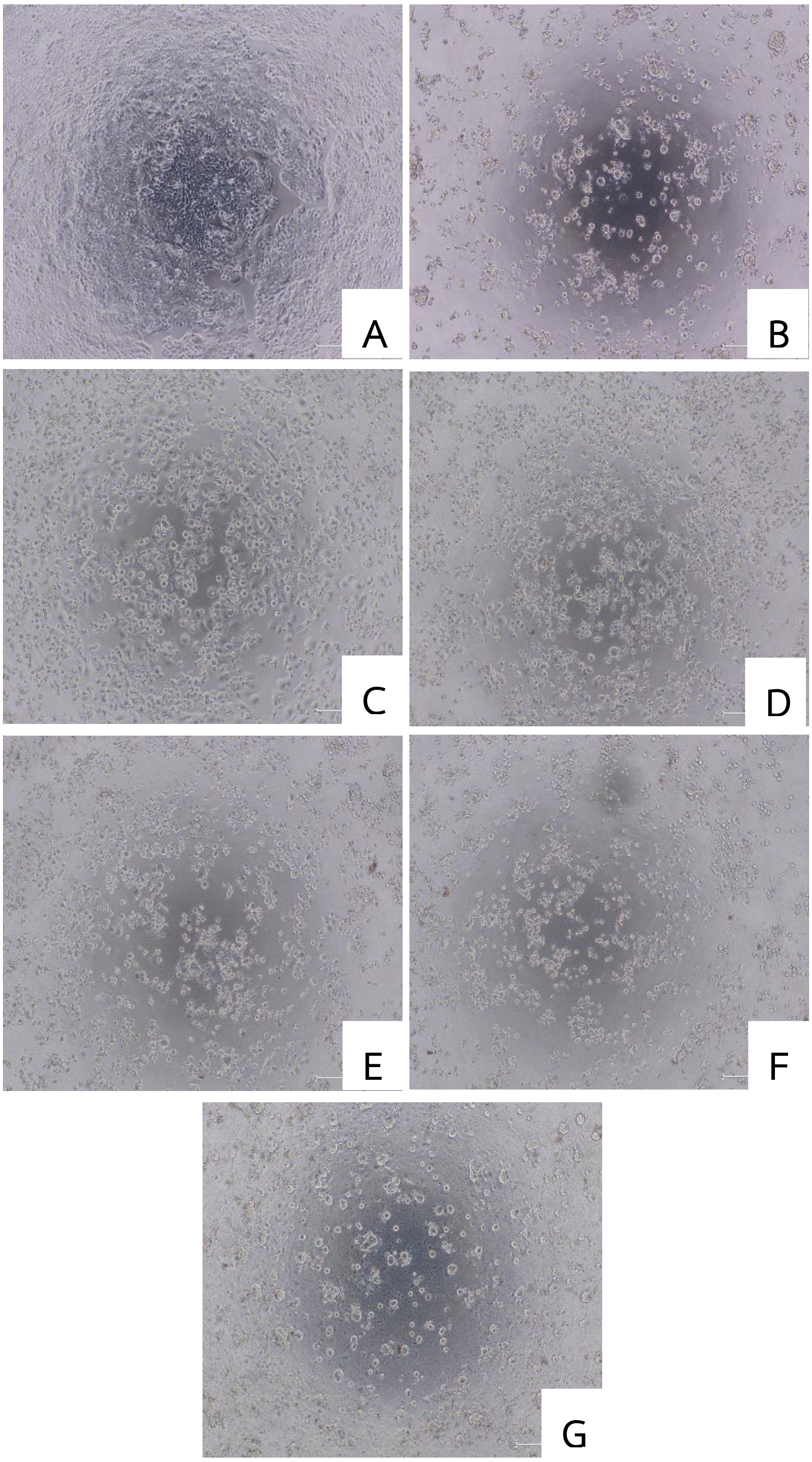

Figure S14. Results of microscopic evaluation of HT-29 cell: [A] untreated cells (negative control), [B] 10 µg mL^{-1} doxorubicin (positive control), [C] CDs-PEG-DOX at 31.25 µg mL^{-1} [D] at 62.5 µg mL^{-1}, [E] at 125 µg mL^{-1}, [F] at 250 µg mL^{-1}, and [G] at 500 µg mL^{-1} examined under the inverted microscope (10× magnification)

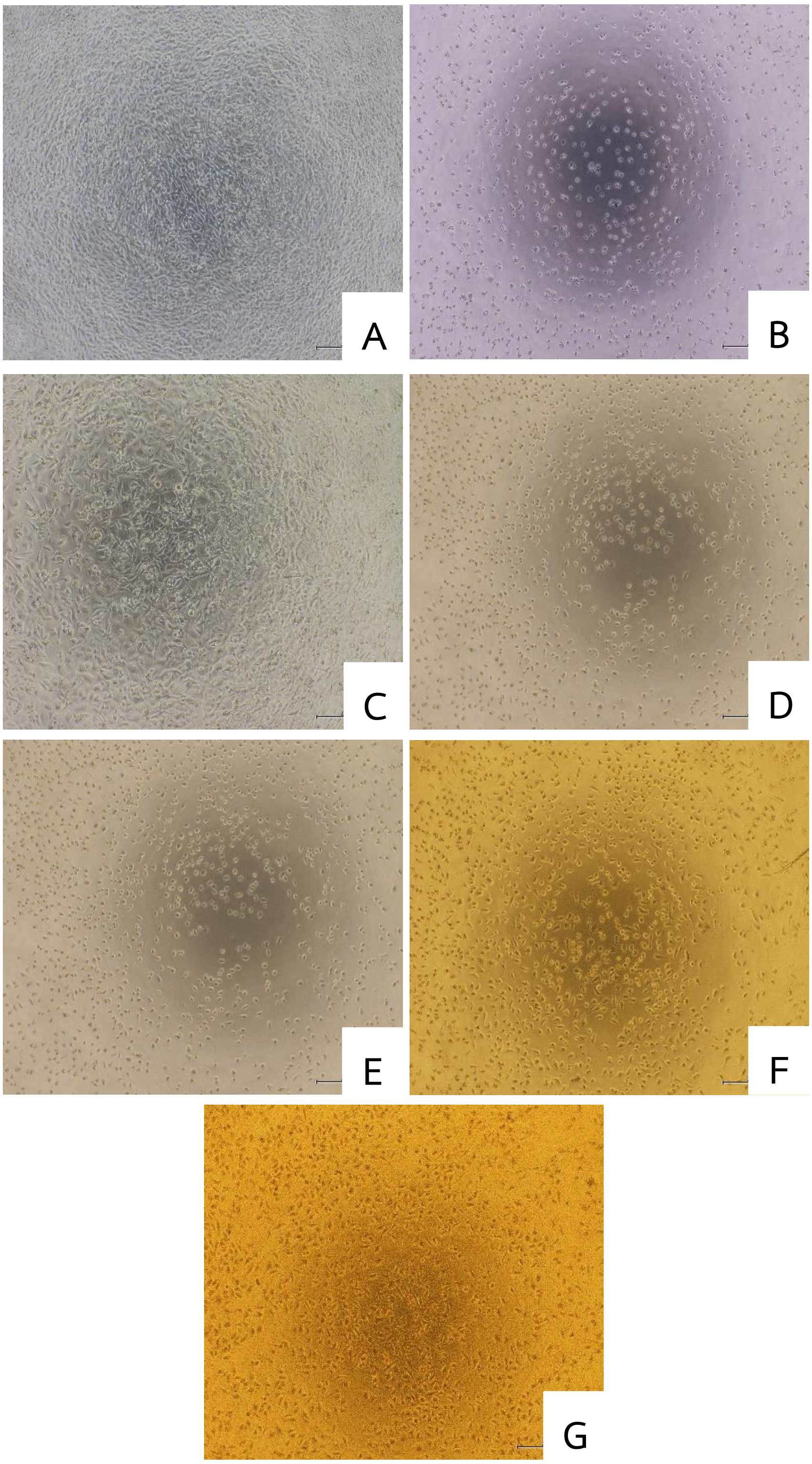

Figure S15. Results of microscopic evaluation of L929 cell: [A] untreated cells (negative control), [B] 10 μg mL^{-1} doxorubicin (positive control), [C] CDs at 625 μg mL^{-1}, [D] at 1250 μg mL^{-1}, [E] at 2500 μg mL^{-1}, [F] at 5000 μg mL^{-1}, and [G] at 10000 μg mL^{-1} examined under the inverted microscope (10× magnification)

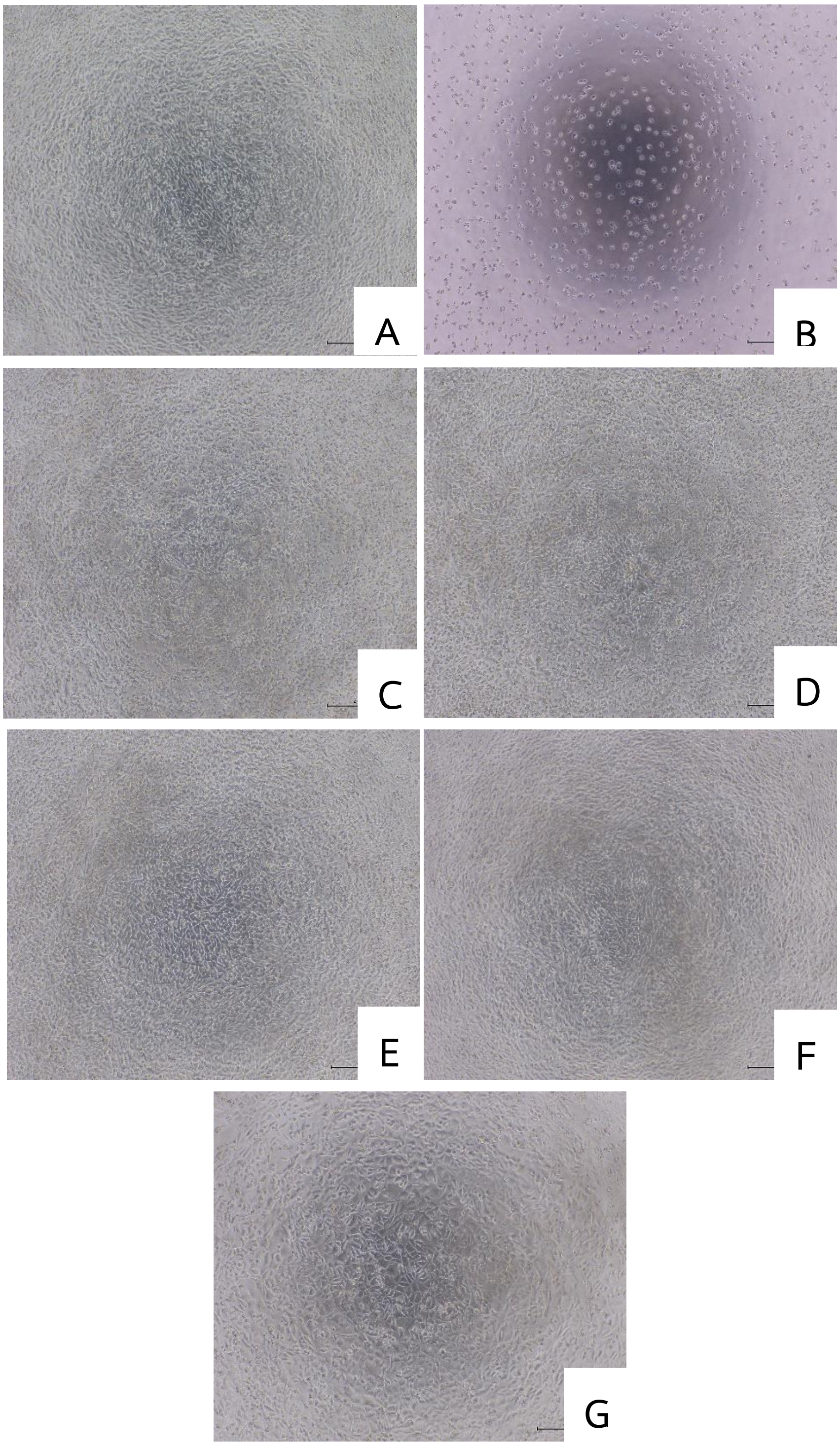

Figure S16. Results of microscopic evaluation of L929 cell: [A] untreated cells (negative control), [B] 10 µg mL^{-1} doxorubicin (positive control), [C] CDs-PEG at 312.5 µg mL^{-1} [D] at 625 µg mL^{-1}, [E] at 1250 µg mL^{-1}, [F] at 2500 µg mL^{-1}, and [G] at 5000 µg mL^{-1} examined under the inverted microscope (10× magnification)

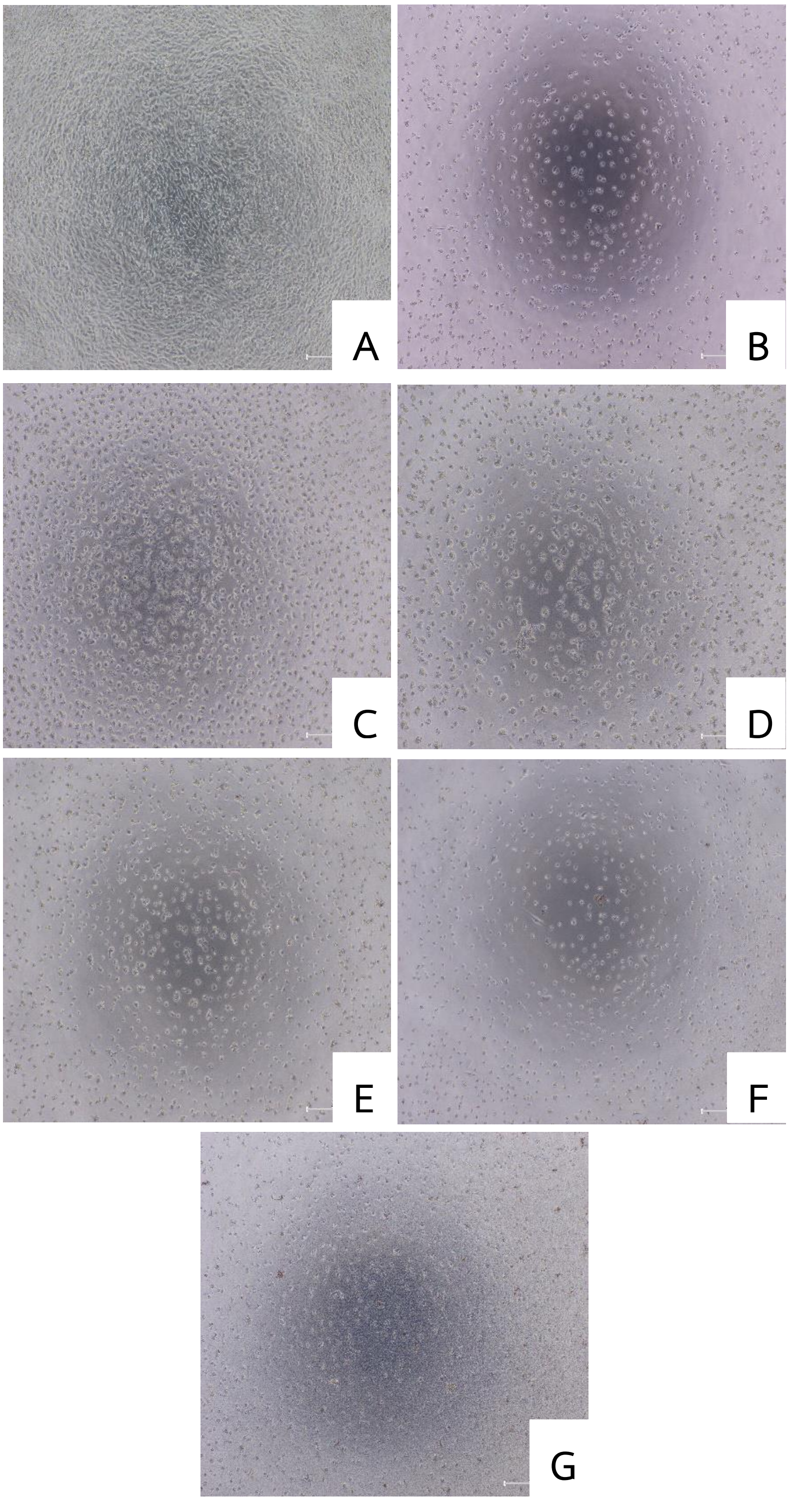

Figure S17. Results of microscopic evaluation of L929 cell: [A] untreated cells (negative control), [B] 10 μg mL^{-1} doxorubicin (positive control), [C] CDs-PEG at 31.25 μg mL^{-1} [D] at 62.5 μg mL^{-1}, [E] at 125 μg mL^{-1}, [F] at 250 μg mL^{-1}, and [G] at 500 μg mL^{-1} examined under the inverted microscope (10× magnification)

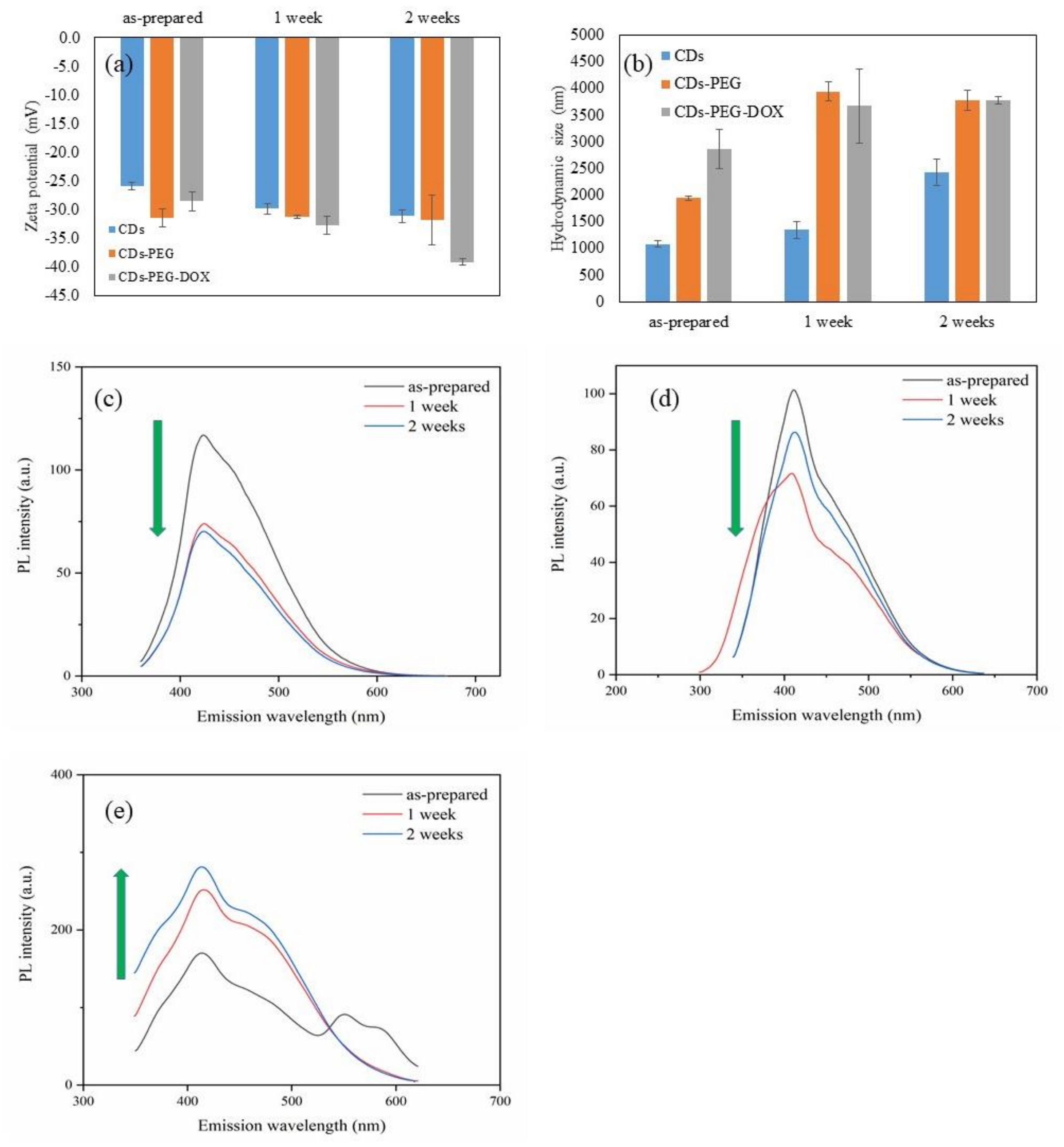

Figure S18. (a) Zeta potential, (b) hydrodynamic diameter, and fluorescence emission spectra of (c) CDs-220C-6h when λ_{Ex} = 350 nm, (d) CDs-PEG-DOX when λ_{Ex} = 330 nm, and (e) CDs-PEG-DOX when λ_{Ex} = 330 nm for the as-prepared sample, and samples stored at 4°C in darkness for 1 week and 2 week.

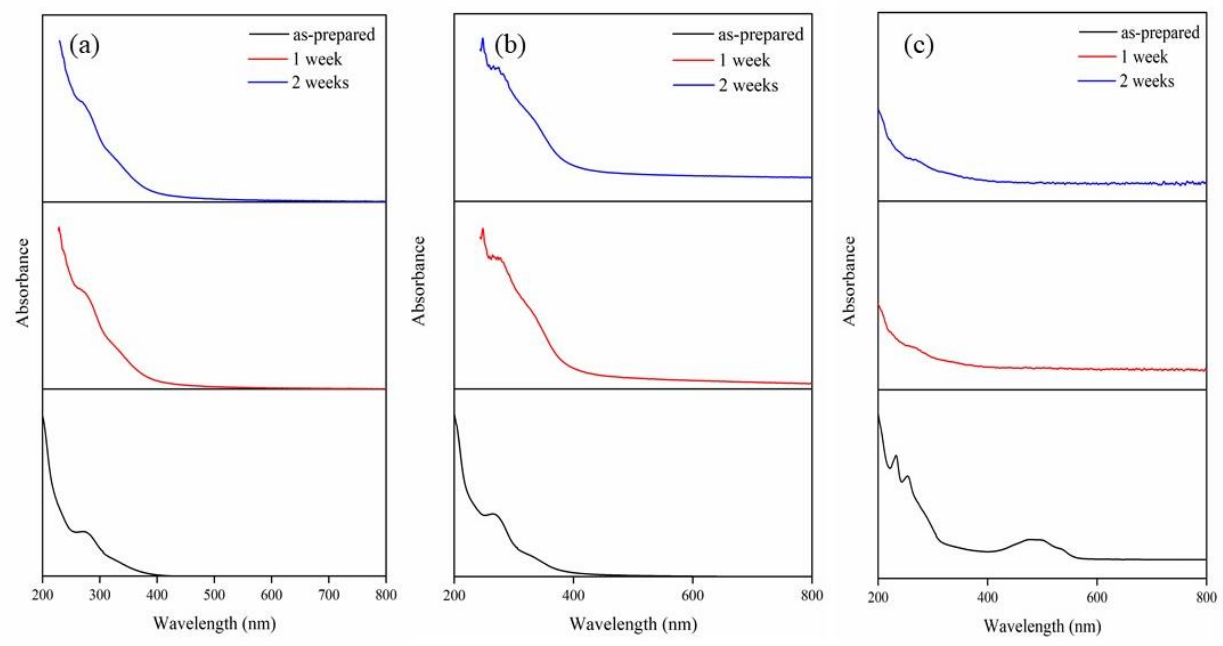

Figure S19. UV-vis spectra of (a) CDs-220C-6h, (b) CDs-PEG, and (c) CDs-PEG-DOX of as-prepared, 1-week and 2-week samples stored at 4°C under darkness.
